# Downregulation of tRNA methyltransferase FTSJ1 by PM2.5 promotes glycolysis and malignancy of NSCLC via facilitating PGK1 expression and translation

**DOI:** 10.1038/s41419-024-07287-0

**Published:** 2024-12-18

**Authors:** Yiling Wang, Yuxin Wen, Qianqian Chen, Yongyi Huang, Duanyang Zhou, Wenhan Yang, Lin Yang, Juan Xiong, Kaiping Gao, Liyuan Sun, Rihong Zhai

**Affiliations:** 1 School of Public Health, Shenzhen University Medical School, 1066 Xueyuan Ave, Shenzhen, 518055 China; 2 Guangdong Provincial Key Laboratory of Genome Stability and Disease Prevention, International Cancer Center, Shenzhen University Medical School, 1066 Xueyuan Ave, Shenzhen, 518055 China; 3 Department of Thoracic Surgery, The People’s Hospital of Shenzhen, 1017 North Dongmen Road, Shenzhen, 518020 China; 4 School of Nursing, Shenzhen University Medical School, 1066 Xueyuan Ave, Shenzhen, 518055 China

**Keywords:** Non-small-cell lung cancer, Cancer metabolism

## Abstract

Fine particulate matter (PM2.5) exposure has been associated with increased incidence and mortality of lung cancer. However, the molecular mechanisms underlying PM2.5 carcinogenicity remain incompletely understood. Here, we identified that PM2.5 suppressed the expression of tRNA methyltransferase FTSJ1 and Am modification level of tRNA in vitro and in vivo. FTSJ1 downregulation enhanced glycolytic metabolism of non-small cell lung cancer (NSCLC) cells, as indicated by increased levels of lactate, pyruvate, and extracellular acidification rate (ECAR). Whereas treatment with glycolytic inhibitor 2-DG reversed this effect. In contrast, upregulation of FTSJ1 significantly suppressed glycolysis of NSCLC cells. Mechanistically, the silencing of FTSJ1 increased NSCLC cell proliferation and glycolysis through enhancing the expression and translation of PGK1. In human NSCLC tumor samples, FTSJ1 expression was negatively correlated with PGK1 expression level and the SUVmax value of PET/CT scan. In summary, our work reveals a previously unrecognized function of PM2.5-downregulated FTSJ1 on PGK1-mediated glycolysis in NSCLC, suggesting that targeted upregulation of FTSJ1 may represent a potential therapeutic strategy for NSCLC.

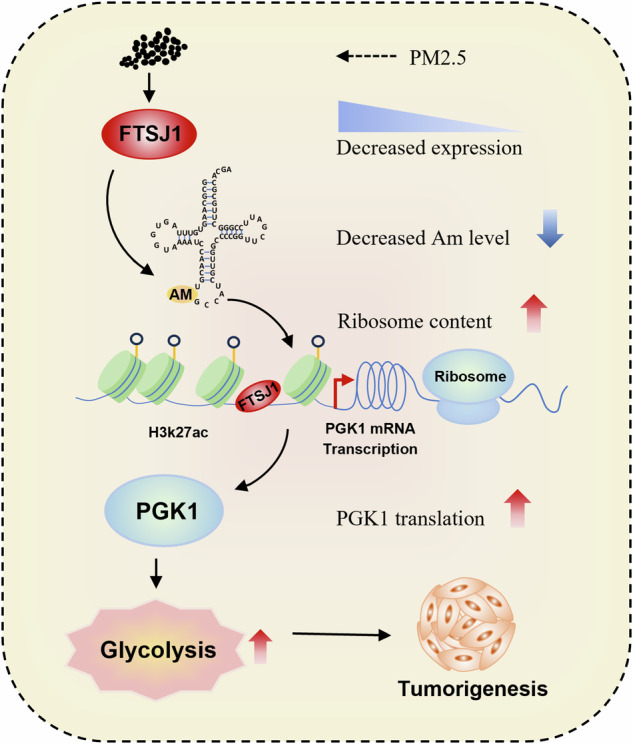

## Introduction

Fine particulate matter (PM2.5) air pollution is a global health problem and ranks as one of the top ten health risk factors in the world [1]. PM2.5 consists of both inorganic (cadmium, nitrate, etc.) and organic (i.e. polycyclic aromatic hydrocarbons, PAHs) carcinogens [2]. Among them, PAHs are the main components, accounting for up to 80% of ambient PM2.5 [3]. Indeed, increasing evidence has revealed that long term exposure to PM2.5 is associated with increased incidence and mortality of lung cancer [4–6]. Multiple meta-analyses have concluded that a 10 µg/m^3^ increase in PM2.5 exposure was related to an increased risk of lung cancer by 9～16% [4, 7]. Therefore, PM2.5 has been categorized as a Class I human carcinogen by the International Agency for Research on Cancer (IARC) [8]. Furthermore, studies have also shown that each 10 µg/m^3^ elevation in PM2.5 air pollution was associated with 8 ~ 27% increase in lung cancer mortality [5, 9–11]. During the past two decades, several potential mechanisms have been proposed for the adverse pulmonary effects of PM2.5, including inflammation, epigenetic modifications, oxidative stress, and DNA damage [12]. For example, it was found that PM2.5 facilitated Wnt5a/JNK pathway-mediated lung inflammation and fibrosis [13]. PM2.5 could induce DNA damage and oxidative stress in human bronchial epithelial cells [14]. Moreover, PM2.5 may induce lung tumorigenesis by regulating methylation level of 15-LOX1/15-LOX2 [15]. Nevertheless, these investigations mainly focus on the acute impacts of PM2.5 on cellular damage. Relatively few studies have concentrated on PM2.5-induced malignant phenotypes and molecular mechanisms in non-small cell lung cancer (NSCLC) cells.

RNA modifications are chemically altered marks on the bases or ribose sugar in RNA molecules [16]. To date, over 170 types of modifications have been found in eukaryotic RNAs [17]. Many of these modifications, particularly m6A, m5C, hm5C, m7G, and m1A, play critical roles not only in modulating gene expression, protein translation, and controlling cell fate [18], but also in regulating cell metabolism [19]. Among different species of RNAs, including mRNA, miRNA, rRNA, and tRNA, tRNA is the most modified type, since more than 85% of all modified nucleosides are present on tRNA molecules [20]. Nevertheless, most previous studies regarding the biological functions and molecular mechanisms of RNA modification in human diseases have focused on mRNA modifications, relatively less attention has been paid to the roles of tRNA modifications in cancers. In recent years, emerging studies have shown that tRNA modifications are crucial for tRNA stability, codon recognition and efficient protein synthesis [17, 21]. Dysregulation of tRNA modification has been associated with various cancers, including lung cancer [22, 23]. However, the underlying mechanisms of tRNA modifications in human cancer are not well understood. The 2’-O-methyladenosine (Am) modification in tRNA is a highly conservative modification found in eukaryotes, prokaryotes, and some archaea [24]. In human, tRNA Am modification is catalyzed by methyltransferase FTSJ1 [25]. Previously, we have found that both the levels of tRNA Am modification and FTSJ1 gene expression were decreased in NSCLC, and downregulation of FTSJ1 promoted the malignant phenotypes of NSCLC cells [26]. But the precise roles and regulatory mechanisms of FTSJ1 in NSCLC are still elusive. It is unknown whether FTSJ1 may be involved in PM2.5-associated carcinogenesis.

In this study, we found that PM2.5 suppressed the expression of FTSJ1 and its downregulation enhanced malignancy and glycolysis reprogramming in NSCLC cells. Mechanistically, FTSJ1 knockdown upregulated the transcription and translation of PGK1, resulting in increased cell proliferation and glycolysis metabolism. Lastly, we verified that lower FTSJ1 expression was negatively correlated with increased glycolysis metabolism and PGK1 expression in patients with NSCLC. Our study uncovers the essential role of the FTSJ1-PGK1-glycolysis axis in PM2.5-associated carcinogenesis and highlights that upregulation of FTSJ1 may be a promising therapy against NSCLC.

## Materials and methods

### Study design

This study was divided into several phases. In the discovery phase, the influences of PM2.5 on FTSJ1 expression and tRNA Am modification were analyzed with qRT-PCR, Western blot (WB), and LC-MS assays in vitro and in vivo. Subsequently, the biological functions and clinical relevance of FTSJ1 in NSCLC were investigated using cellular, molecular and biochemical experiments in NSCLC cells and in NSCLC clinical samples. Then, the downstream target gene of FTSJ1 was identified and validated by RNA-seq, qRT-PCR, biochemical, and rescue assays. Lastly, the regulatory mechanisms of FTSJ1 on target gene were explored by polysome profiling, WB, qRT-PCR, and Chromatin immunoprecipitation (ChIP) assays.

### PM2.5 sample preparation

The procedures for PM2.5 sample preparation and exposure dosage assessments have been described in our previous report [27]. Briefly, an urban air PM2.5 sample (SRM 1648a, encoded as PM2.5) with detailed constituent information was obtained from the National Institute of Standards and Technology (NIST, MD, USA). The major components of this PM2.5 are PAHs and nitro-PAHs (NIST Certificate of Analysis, 2020). PM2.5 powder was suspended in phosphate buffer saline (PBS) and sonicated for 20 min before being used for experiments.

### Clinical specimens

This study was approved by the Medical Ethics Committee of Shenzhen University (Approved no. 2016002). A total of 19 tumor tissues were collected from NSCLC patients who underwent surgical resection in the Department of Thoracic Surgery at The People’s Hospital of Shenzhen, China (Table S1). Sample size was estimated using the PS software (http://biostat.mc.vanderbilt.edu/PowerSampleSize). Assumed that α = 0.05 (t-test), β = 90%, SUVmax index change≥0.2, and standard deviation = 0.15; under these conditions, 13 samples could obtain a power of 90%. Our study included 19 samples, meeting the statistical rigor to interpret the data with confidence. Written informed consents were obtained from all patients before sample collection. The stage classification was defined according to the criteria of the Eighth Edition Lung Cancer Stage Classification (IASLC) [28]. No patients received any kind of therapy or had a history of other malignancies prior to surgery. After surgical resection, tumor samples were fixed in formalin, embedded in paraffin, and then stored for further processing.

### Cell culture and PM2.5 treatment

NSCLC cell lines (A549, H358) and normal human pulmonary epithelial cell line (BEAS-2B) were purchased from Cell Resource Center, Shanghai Institute for Biological Sciences (Shanghai, China). The short tandem repeat (STR) assay was used to verify the identity of all cell lines. The absence of mycoplasma contamination was tested by PCR-based ELISA assay. A549 and BEAS-2B cells were cultured in DMEM medium (cat# 11965092, Gibco, NY, USA) containing 10% FBS (cat# A5670701, Gibco, NY, USA). H358 cells were incubated in RPMI-1640 basic (1×) medium (Gibco, cat# 11875085, NY, USA). All cells were cultured in a humidified incubator with 5% CO_2_ at 37 °C. Based on cell viability experiments reported by us and others in previous studies [27, 29], cells were cultured under different concentrations of PM2.5 (0, 25, 50, 100, 200, 400 μg/ml), respectively. All cell culture experiments were performed in triplicate.

### Animal experiments for PM2.5 exposure

Specific pathogenfree (SPF) male Sprague-Dawley (SD) rats (4 weeks old, 200 g) were purchased from Guangdong Experimental Animal Center (Foshan, China). After one week of acclimation, rats were randomly divided into 4 groups (n = 5 per group) using a random number table and the investigator was blinded to the group allocation during the experiments. The sample size of animals and experimental doses of PM2.5 were estimated according to previous studies [27, 30]. Briefly, the WHO recommended mean concentration of interim target 1 (35 µg/m^3^ of PM2.5) was used as the basis of ambient concentration to determine the inhaled amount of PM2.5. After considering the respiratory parameter of rats, the inhaled PM2.5 amount was converted to PM2.5 doses as 1.8, 5.4, and 16.2 mg/kg body weight for intratracheal instillation.

**Fig. 1:**
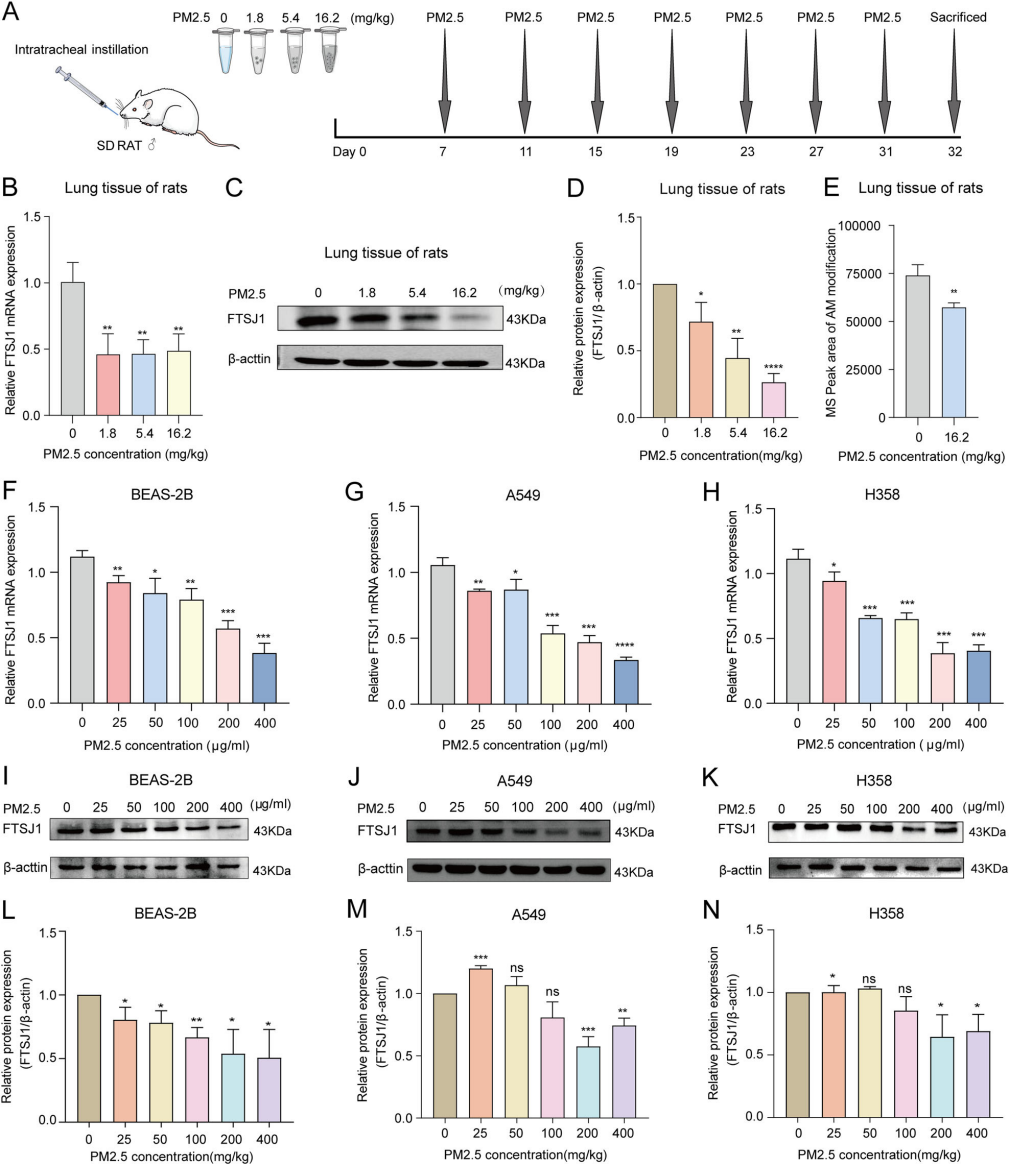
PM2.5 suppresses the expression of tRNA methyltransferase FTSJ1 in vitro and in vivo. **A** Schematic diagram and timeframe for PM2.5 exposure experiments in rat model. **B** PM2.5 inhibits FTSJ1 gene expression in lung tissues of rats. **C**, **D** PM2.5 suppresses FTSJ1 protein expression in lung tissue of rats in a dose-response manner. **E** PM2.5 downregulates tRNA AM modification levels in lung tissue of rats. **F** PM2.5 inhibits FTSJ1 gene expression in BEAS-2B cells. **G** PM2.5 suppresses FTSJ1 gene expression in A549 cells. **H** PM2.5 reduces FTSJ1 gene expression level in H358 cells. **I**, **L** PM2.5 downregulates FTSJ1 protein expression in BEAS-2B cells. **J**, **M** PM2.5 inhibits FTSJ1 protein expression in A549 cells. **K**, **N** PM2.5 suppresses FTSJ1 protein expression in H358 cells. Data are presented as mean ± SD, Student’s *t* test. NS not significant; *p < 0.05 vs. control (no PM2.5 exposure); **p < 0.01 vs. control；***p < 0.001 vs. control; ****p < 0.0001 vs. control.

All rats were anesthetized by intraperitoneal injection of 5% chloraldurate (Sigma, cat# 23120). For the PM2.5-treated groups, animals were intratracheally instilled with PM2.5 suspensions every 3 days for 24 days (Fig. 1A). The control group was treated with an equal volume normal saline in the same manner as the PM2.5-treated groups. At the end of the experiment, the rats were euthanized, and lung tissue was extracted and stored at −80 °C for subsequent analysis. All animal experiments were carried out following the procedures approved by the Institutional Animal Care and Use Committee of Shenzhen University Medical School (Approval No. IACUC-202300059).

### tRNA isolation and LC-MS analysis

Total RNA was extracted from lung tissues of rats using Trizol reagent (cat# 15596026CN, Invitrogen, Shanghai, China). tRNA was isolated from total RNA by 7.5% PAGE electrophoresis and size selection. The purified tRNAs were hydrolyzed to mononucleosides and then dephosphorylated with a mixture of 10U benzoase, 0.1U phosphodiesterase I, and 1U alkaline phosphatase, respectively. tRNA modification was analyzed by liquid chromatography-coupled mass spectrometry (LC-MS) using the 6460 QQQ mass spectrometer (Agilent Technologies, Inc, CA, USA) with an Agilent 1290 HPLC system. The peak information of nucleoside was extracted by Agilent qualitative analysis software. A peak signal-to-noise ratio of 10 or more is considered a detectable nucleoside. The peak area was then normalized to the number of purified tRNAs per sample.

### Cell transfection

FTSJ1-overexpression (OE) plasmid and the empty negative control (NC) were constructed using the pcDNA3.1 (+) vectors by GenePharma (Suzhou, China). siRNAs for knockdown of FTSJ1 and siRNA-NC were designed and synthesized by GenePharma (Suzhou, China) too. Transfection efficiencies of OE-plasmids and siRNAs were verified by Western blot and qRT-PCR assays (Fig. S1). Cell transfection was performed using the Lipofectamine 3000 reagents (Invitrogen, cat# L3000015, Shanghai, China) according to the manufacturer’s protocol. All in vitro experiments were performed in triplicate.

### RNA-seq

PC9 cells were transfected with FTSJ1 overexpression plasmids or NC respectively. Total RNAs were extracted from cells using the TRIzol reagents (Invitrogen, Shanghai, China). NEBNext® Poly(A) mRNA Magnetic Isolation Module (cat# E3370, New England Biolabs, MA, USA) was used to isolate intact poly(A) + RNA from the total RNA. KAPA Stranded RNA-Seq Library Prep Kit (Illumina, CA, USA) was applied to prepare the libraries, which were further sequenced on the Illumina HiSeq 4000 (PE150) by KangChen Biotech Company (Shanghai, China). FPKM (Fragments per Kilobase of transcript per Million mapped reads) was estimated using the ballgown (2.10.0) software and DESeq2 was used for calculating gene expression levels.

### Immunohistochemistry (IHC) analysis

IHC was performed as previously described [31]. Anti-FTSJ1 (Atlas, HPA002718), anti-PGK1 (Proteintech, 17811-1-AP) antibodies were used for IHC assay. Images were observed and analyzed under microscope (ZEISS, Axio lab. A1) using Image J software. The intensity of immunoreactive staining was defined as: 0 (no staining), 1 (weak staining), 2 (moderate staining), and 3 (strong staining). The proportion of positive staining cells was graded as: 0 (0% positive cells), 1 ( ≤ 25% positive cells), 2 (26-50% positive cells), 3 (51-75% positive cells), and 4 (>75% positive cells), respectively. The final IHC score was determined by multiplying the positive extent and staining intensity. The IHC score ≦3 (median value) was defined as low-expression, and score>3 was graded as high-expression.

### Quantification of lactate and pyruvate

In brief, transfected cells (1 × 10^6^) were inoculated into six-well plates for 24 h. The supernatant of culture medium was collected, and the L-lactate assay kit (A019–2-1, Jiancheng, Nanjing, China) and pyruvate assay kit (A019–2-1, Jiancheng, Nanjing, China) were used to measure the production of lactate and pyruvate from the cells, respectively. The concentration of lactate or pyruvate in each sample was calculated via standard curve calibration.

### Extracellular acidification and oxygen consumption rate assays

The extracellular acidification rate (ECAR) and cellular oxygen consumption rate (OCR) of cells were determined using the Seahorse XFe24 Flux Analyzer (Seahorse Biosciences, Agilent). Experiments were performed according to the manufacturer’s protocols. ECAR and OCR were examined using the Seahorse XF Glycolysis Stress Test Kit (cat #103020–100) and Seahorse XF Cell Mito Stress Test Kit (cat # 103015–100), respectively. Briefly, 5 × 10^4^ cells per well were seeded into a Seahorse XF 24 cell culture microplate and cultured in XF base medium (pH 7.4) at 37 °C overnight. For ECAR, glucose (10 mM), glutamine (1 mM), 2-DG (50 mM) and oligomycin (1 μM) were added sequentially into the plates at specific time points following the manufacturer’s guidelines. For OCR, oligomycin, the reversible inhibitor of oxidative phosphorylation FCCP (p-trifluoromethoxy carbonyl cyanide phenylhydrazone), and the mitochondrial complex I inhibitor rotenone plus the mitochondrial complex III inhibitor antimycin A (Rote/AA) were sequentially injected into the wells. Data of OCR and ECAR were collected and analyzed using the Seahorse XF24 software.

### Western blotting

Total proteins from cells or tissues were extracted using the RIPA lysis buffer (cat# 89900, Promega, Madison, USA) and measured with BCA method. Proteins were separated on an 12% SDS-PAGE gel and then transferred to PVDF membranes (Millipore, cat# ISEQ00010). The membrane was blocked with 5% BSA at room temperature for 1 h and then incubated with primary antibodies at 4 °C overnight, followed by incubating with horseradish peroxidase (HRP) conjugated at room temperature for 90 min. The protein bands were scanned and visualized by the GS700 imaging densitometer (Bio-Rad Laboratories) and analyzed by Image J software.

### Quantitative real-time PCR (qRT-PCR)

TRIzol reagent (cat# 15596026, Invitrogen, Shanghai, China) was used to extract total RNA from tissues and cells according to the manufacturer’s protocol. RNA was reversely transcribed into complementary DNA (cDNA) using the PrimeScript™RT reagent Kit with gDNA Eraser (cat# RR047A, Takara, Tokyo, Japan). TB Green Premix Ex Taq (cat# RR820A, Takara, Tokyo, Japan) was used to amplify transcription levels of specific genes and standardized them to ACTB. The relative expression was calculated using the 2^−ΔΔCt^ method. The primer sequences are listed in Table S2.

### Polysome profiling

Isolation of ribosome-bound mRNA by polysome fractionation was performed as previously described with minor modifications [27]. Briefly, A549 cells were transfected with FTSJ1-overexpression plasmids, si-FTSJ1, and their corresponding NCs, respectively. Before collection, cells were washed by PBS (containing 100 μg/mL cycloheximide) for 10 min. Then cells were trypsinized at 37 °C for 3 min and then treated with multimer extraction buffer (PEB; 20 mM Tris-HCl, pH 7.5, 50 mM KCl; MgCl2 10 mm; 1 mM DTT; 100 μg/ml chlorhexidine; 200 μg/ml heparin) containing 1% Triton-X100. Cell lysates were centrifuged at 14,000 rpm for 30 min at 4 °C. The cell supernatant was transferred onto the top of 5-50% sucrose density gradient solution and centrifuged at 38000 rpm for 2 h in an Optima L-100XP rotor (Beckman Coulter). Gradient fractions were collected using a Piston Gradient Fractionator Station (BioComp Instruments) with continuous monitoring of absorbance at 260 nm. RNA fractions 1-17 were pooled together as light weight fraction, and RNA samples extracted from fraction 19-23 were merged as heavy weight fraction of the ribosome [32]. The corresponding isolated fractions were further used for RNA extraction with TRIzol reagents (Life Tech., Shanghai, China). The extracted RNA samples were then subject to qRT-PCR analysis.

### Cell proliferation assay

Transfected cells were seeded in 96-well plates at a density of 3000 cells/well. The cell proliferation was analyzed using the CCK8 assay kit (Dojindo, cat# ck04, Osaka, Japan) at 0, 24, 48, 72, and 96 h time point after the cells were seeded. A volume of 10 µl CCK8 reagent was added to each well. After incubation for 1 h, the plates were read at a wavelength of 450 nm to measure the absorbance of each well using a Synergy HTX multimode microplate reader (BioTek, Guangzhou, China). Three replicate wells were tested for each group at the same time point.

### Chromatin immunoprecipitation (ChIP) assay

ChIP assay was performed with a ChIP assay kit (Thermo Scientific, cat# 26156) according to the manufacturer’s protocol. Briefly, A549 cells were cross-linked with formaldehyde, lysed and sonicated to an average size of 200-to-500 fragments. Cell lysates were precleared with protein A/G beads and immunoprecipitation was performed using anti-FTSJ1 antibody (Santa Cruz, sc-390355) and IgG. The precipitated DNA was subjected to real-time PCR. The specific primers used for ChIP-qPCR are presented in Table S2.

### Statistical analysis

All statistical analyses were performed using Graph Pad Prism 6 (Graph Pad, USA) and SAS 9.4 (SAS Institute, USA) software. Quantitative data were presented as means ± SD from at least three independent experiments. Normal distribution of data was tested using the Normality and Lognormality tests function in Graph Pad Prism. Variance between comparing groups was estimated by Levene test. Comparisons between groups were made using the Student’s t-test (for parametric data) or the Mann-Whitney test (for non-parametric data). *p* value (bilateral) <0.05 was considered statistically significant.

## Results

### PM2.5 suppresses the expression of FTSJ1 in vitro and in vivo

To investigate the impact of PM2.5 on FTSJ1 gene expression, we first analyzed the expression of FTSJ1 in lung tissues of PM2.5-exposed rats. We found that the mRNA (Fig. 1B) and protein (Fig. 1C, D) levels of FTSJ1 in PM2.5-exposed lung tissues were significantly lower than that of control tissues. Interestingly, the levels of tRNA Am modification, which is catalyzed by FTSJ1, in lung tissues of PM2.5-exposed rats were also lower than that of control tissues (Fig. 1E). In human cells (BEAS-2B, A549, and H358) treated with PM2.5, the expression levels of FTSJ1 significantly decreased in response to PM2.5 exposure levels in a dose-response manner, with the highest FTSJ1 expression level seen in the lowest PM2.5 exposure cells (25 μg/ml) and the lowest FTSJ1 expression level observed in the highest PM2.5 exposure cells (400 μg/ml) (Fig. 1F–H). Similarly, FTSJ1 protein expression levels were downregulated in PM2.5-exposed cells (Fig. 1I–N). These findings indicate that PM2.5 could suppress FTSJ1 expression and decrease tRNA Am modification levels.

### PM2.5 accelerates glycolysis metabolism in BEAS-2B and NSCLC cells

**Fig. 2:**
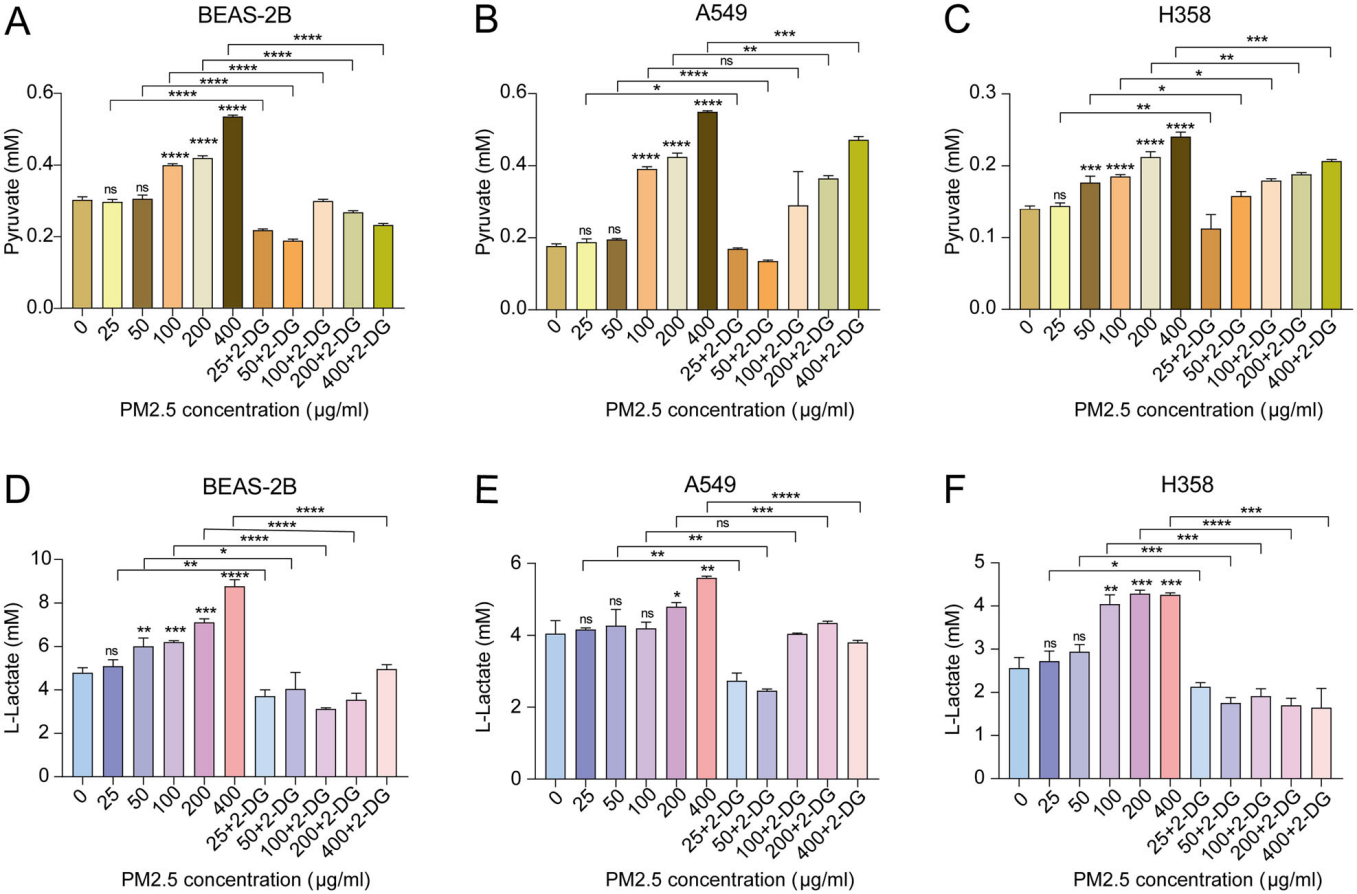
PM2.5 promotes glycolysis metabolism in BEAS-2B and NSCLC cells. **A** PM2.5 increases the production of pyruvate from BEAS-2B cells, and the effect of PM2.5 on pyruvate production is suppressed by 2-DG. **B** PM2.5 enhances the generation of pyruvate from A549 cells in a dose response fashion, and 2-DG treatment alleviates the impacts of PM2.5 on pyruvate generation from A549 cells. **C** PM2.5 promotes pyruvate release from H358 cells, and 2-DG inhibits the effects of PM2.5 on pyruvate release from H358 cells. **D** PM2.5 augments L-lactate release from BEAS-2B cells, and 2-DG suppresses the effects of PM2.5 on L-lactate release from BEAS-2B cells. **E** PM2.5 up-regulates L-lactate levels in the culture medium A549 cells, while 2-DG downregulates the levels of PM2.5-induced L-lactate. **F** PM2.5 augments the production of L-lactate from H358 cells, and 2-DG represses PM2.5-induced L-lactate production from H358 cells. Data are presented as mean ± SD, Student’s *t* test. NS not significant; *p < 0.05, **p < 0.01, ***p < 0.001. All *p* values are versus control or similar PM2.5 exposure level group.

Previously, we have found that PM2.5 exposures at high concentrations (100, 200, 400 μg/ml) significantly enhanced glycolysis metabolism in BEAS-2B cells and in NSCLC cells (A549, PC9) [19]. In the present study, we extended these analyses to investigate the impact of lower PM2.5 levels (25, 50 μg/ml) on glycolysis metabolism in A549 and H358 cells. The pyruvate production analysis demonstrated that PM2.5 exposure promoted the lactate accumulation in a dose-response fashion in both BEAS-2B and NSCLC cells (A549, H358) (Fig. 2A–C). The L-lactate assay also showed that higher PM2.5 concentrations resulted in more L-lactate production from cells (Fig. 2D–F). Conversely, 2-DG (inhibitor of glycolysis) treatment significantly blocked PM2.5-induced L-lactate and pyruvate production in all tested cells (BEAS-2B, A549, H358). Taken together, these data demonstrate that PM2.5 promotes glycolysis metabolism in BEAS-2B and NSCLC cells.

### Downregulation of FTSJ1 enhances glycolysis metabolism in NSCLC cells

**Fig. 3:**
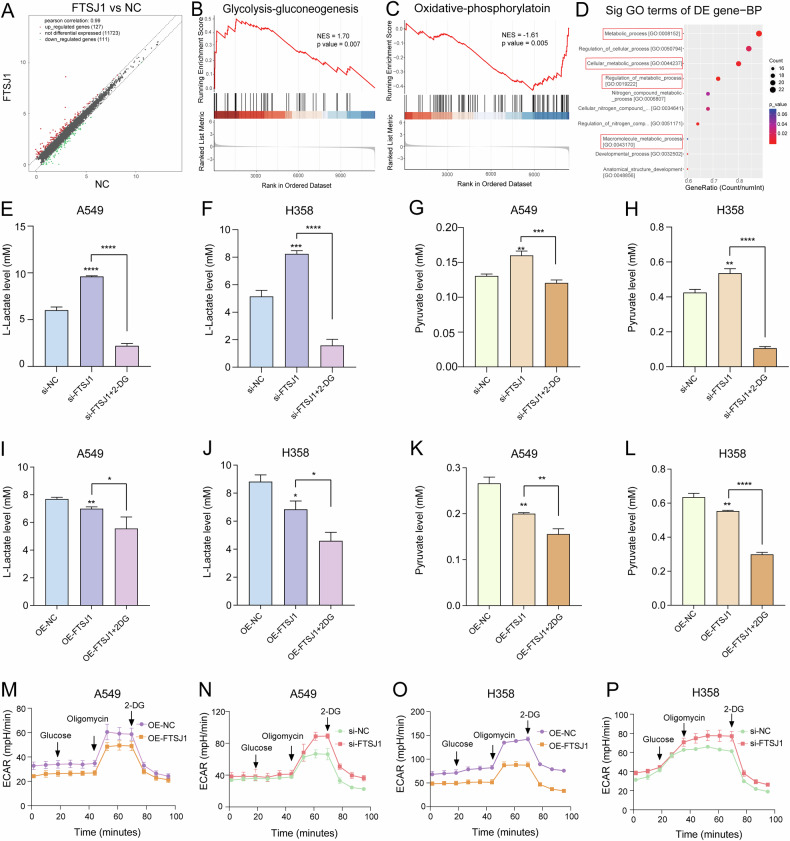
Down-regulation of FTSJ1 increases glycolysis metabolism in NSCLC cells. **A** FTSJ1 induces differentially expressed genes (DEGs) in NSCLC cells. **B** GSEA suggests that DEGs are enriched in glycolysis-gluconeogenesis pathway. **C** DEGs are abundant in oxidative phosphorylation pathway. **D** GO analysis implies that DEGs are involved in metabolic biological processes. **E** Downregulation of FTSJ1 promotes L-lactate production in A549 cells. **F** Inhibition of FTSJ1 leads to higher generation of L-lactate from H358 cells. **G** Knockdown of FTSJ1 potentiates production of pyruvate in A549 cells. **H** Suppression of FTSJ1 promotes pyruvate generation from H358 cells. **I** Upregulation of FTSJ1 suppresses L-lactate production in A549 cells. **J** Overexpression of FTSJ1 inhibits L-lactate release from H358 cells. **K** Higher FTSJ1 expression decreases pyruvate production in A549 cells. **L** Increased expression of FTSJ1 reduces the production of pyruvate from H358 cells. **M** Overexpression of FTSJ1 decreases the levels of extracellular acid ratio (ECAR) in A549 cells. **N** Downregulation of FTSJ1 increases ECAR levels of A549 cells. **O** FTSJ1 suppresses ECAR levels of H358 cells. **P** Inhibition of FTSJ1 enhances ECAR levels of H358 cells. Data are presented as mean ± SD, Student’s *t* test. *p < 0.05, **p < 0.01, ***p < 0.001; ****p < 0.0001. All *p* values are versus NC or between different treated groups.

To explore the biological functions of FTSJ1 on NSCLC, we compared the transcriptome profiles between FTSJ1-overexpressing and control A549 cells. We found that FTSJ1 induced a number of differentially expressed genes (DEGs) (Fig. 3A). Gene Set Enrichment Analysis (GSEA) revealed that FTSJ1-induced DEGs were significantly enriched in oxidative phosphorylation and glycolysis-gluconeogenesis pathways (Fig. 3B, C). Gene ontology (GO) analysis on DEGs showed that FTSJ1 appeared to regulate genes involved in metabolic pathways (Fig. 3D). These findings suggest that FTSJ1 may be implicated in glucose metabolism in NSCLC cells.

To determine whether FTSJ1 may play a role in glycolysis metabolism of NSCLC cells, we measured the levels of lactate and pyruvate, the end metabolites of glycolysis, in the medium of cell culture. As can be seen in Fig. 3, knockdown of FTSJ1 significantly increased the production of L-lactate and pyruvate from both A549 and H358 cells (Fig. 3E–H). In contrast, overexpression of FTSJ1 dramatically decreased L-lactate and pyruvate release from NSCLC cells (Fig. 3I–L). On the other hand, 2-DG treatment significantly blocked FTSJ1-induced L-lactate and pyruvate productions in A549 and H358 cells. In addition, overexpression of FTSJ1 decreased extracellular acidification rate (ECAR), an indicator of overall glycolytic flux, in NSCLC cells (Fig. 3M, O). Whereas knockdown of FTSJ1 ECAR levels in both A549 and H358 cells (Fig. 3N, P). Notably, upregulating of FTSJ1 increased the oxygen consumption rate (OCR) of A549 cells (Fig. S2). In summary, these data suggest that PM2.5-induced downregulation of FTSJ1 may contribute to carcinogenesis through enhancing glycolysis metabolism in NSCLC cells.

### Glycolytic gene PGK1 is a direct target of FTSJ1

**Fig. 4:**
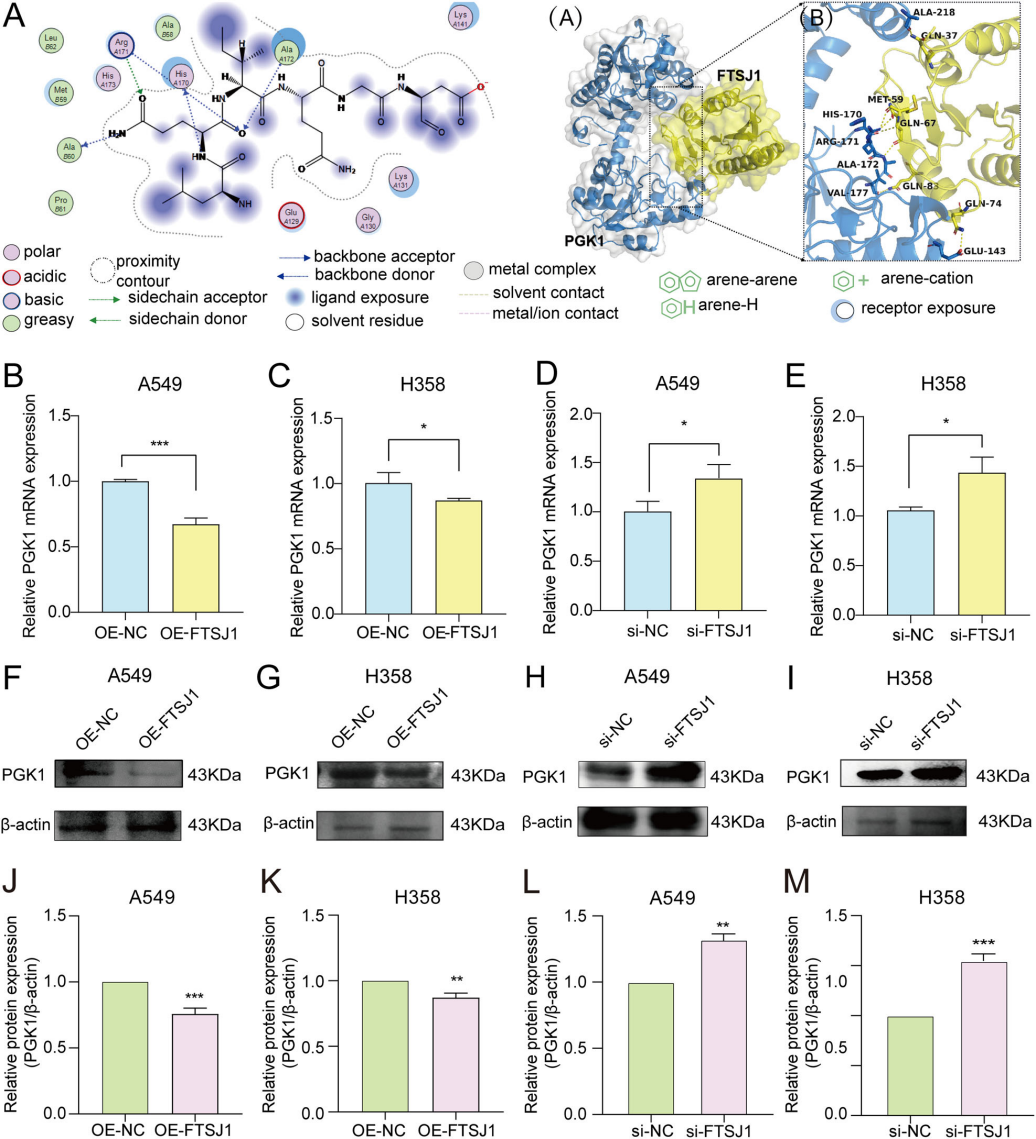
FTSJ1 modulates the expression of glycolytic gene PGK1 in NSCLC cells. **A** Molecular structure of FTSJ1 (left) and predicted 3D structures of FTSJ1 and PGK1, and the binding mode of FTSJ1 to PGK1 (right). **B** Overexpression of FTSJ1 suppresses PGK1 gene expression in A549 cells. **C** Up-regulation of FTSJ1 inhibits PGK1 gene expression in H358 cells. **D** Inhibition of FTSJ1 expression promotes PGK1 gene expression in A549 cells. **E** Knockdown of FTSJ1 enhances PGK1 gene expression in H358 cells. **F**, **J** Overexpression of FTSJ1 suppresses PGK1 protein expression in A549 cells. **G**, **K** Upregulation of FTSJ1 downregulates PGK1 protein expression in H358 cells. **H**, **L** Downregulation of FTSJ1 increases PGK1 protein expression in A549 cells. **I**, **M** Knockdown of FTSJ1 promotes PGK1 protein expression in H358 cells. Data are presented as mean ± SD, Student’s *t* test. *p < 0.05, **p < 0.01, ***p < 0.001. All *p* values are versus NC.

To explore the molecular mechanisms of FTSJ1 in the regulation of aerobic glycolysis, we reanalyzed our previous RNA-seq and Ribo-seq data in which we found that translation efficiency (TE) of PM2.5-regulated genes were enriched in glycolysis pathways [27]. Among the top-scoring glycolysis-related genes, the TEs of LDHA (a key enzyme of the last step of glycolysis), PGK1 and PKM2 (the only 2 enzymes that catalyze ATP production during aerobic glycolysis) (Fig. S3A), were upregulated by PM2.5 exposure. Furthermore, qRT-PCR assays on key glycolytic genes showed that up-regulation of FTSJ1 markedly suppressed the expression of PGK1 and LDHA (Fig. S3B, C). In contrast, knockdown of FTSJ1 significantly enhanced the expression level of PGK1 and LDHA (Fig. S3E, F). Nevertheless, the impacts of FTSJ1-overexpression on PGM2 and PKM expressions were statistically not significant (Fig. S3D, G). As can be seen from Fig. S3, the impact of FTSJ1 on PGK1 expression seemed more statistically significant than that of LDHA. Moreover, molecular docking analysis showed that several amino acid residues in FTSJ1 protein could bind to PGK1 (Fig. 4A). In addition, both qRT-PCR and Western blot assays confirmed that FTSJ1 overexpression suppressed the protein expression levels of PGK1 (Fig. 4B, C, F, G, J, K). While knockdown of FTSJ1 increased the protein expression levels of PGK1 (Fig. 4D, E, H, I, L, M). These findings suggest that PGK1 may act as the major target of FTSJ1 in PM2.5-induced carcinogenesis.

### FTSJ1 regulates cell proliferation and glycolysis metabolism of NSCLC cells in a PGK1-dependent manner

**Fig. 5:**
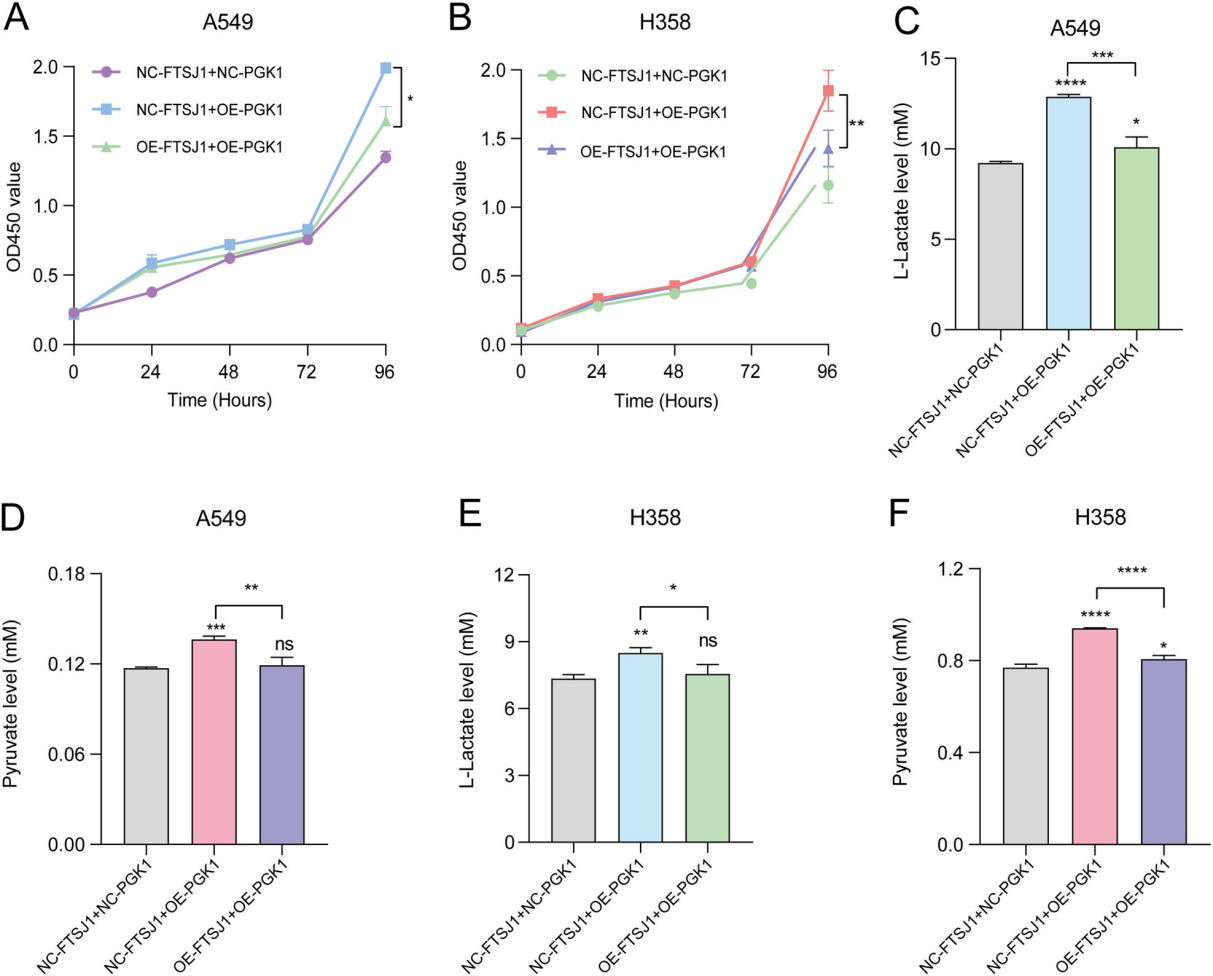
FTSJ1 regulates aerobic glycolysis and proliferation of NSCLC cells in a PGK1-dependent manner. **A** Rescue assays showed that co-transfection of OE-FTSJ1 and OE-PGK1 suppresses the promoting ability of PGK1 on proliferation of A549 cells. **B** Up-regulation of FTSJ1 decreases PGK1-induced proliferation rate in H358 cells. **C** OE-FTSJ1 inhibits PGK1-induced production of L-Lactate from A549 cells. **D** OE-FTSJ1 reduces PGK1-mediated production of pyruvate from A549 cells. **E** OE-FTSJ1 decreases PGK1-induced release of L-Lactate from H358 cells. **F** OE-FTSJ1 inhibits PGK1-mediated generation of pyruvate from H358 cells. Data are presented as mean ± SD, Student’s *t* test. *p < 0.05, **p < 0.01, ***p < 0.001; ****p < 0.0001. All *p* values are versus NC or between different treated groups.

To examine whether PGK1 could mediate the biological functions of FTSJ1 in NSCLC cells, a series of rescue experiments were executed by co-transfecting OE-PGK1 and OE-FTSJ1 into NSCLC cells. The results showed that overexpression of FTSJ1 significantly reversed the promoting effects of OE-PGK1 in cell proliferation in both A549 and H358 cells (Fig. 5A, B). In glycolysis metabolism assays, up-regulation of FTSJ1 dramatically blocked PGK1-induced L-lactate and pyruvate productions from A549 cells (Fig. 5C, D). Similarly, overexpression of FTSJ1 significantly suppressed the impact of PGK1 on L-lactate and pyruvate generations from H358 cells (Fig. 5E, F). Taken together, these data demonstrate that FTSJ1 regulates glycolytic metabolism and cell proliferation through interactions, at least in part, with PGK1 in NSCLC cells.

### FTSJ1 regulates the translation of PGK1 mRNA in the ribosomes of NSCLC cells

**Fig. 6:**
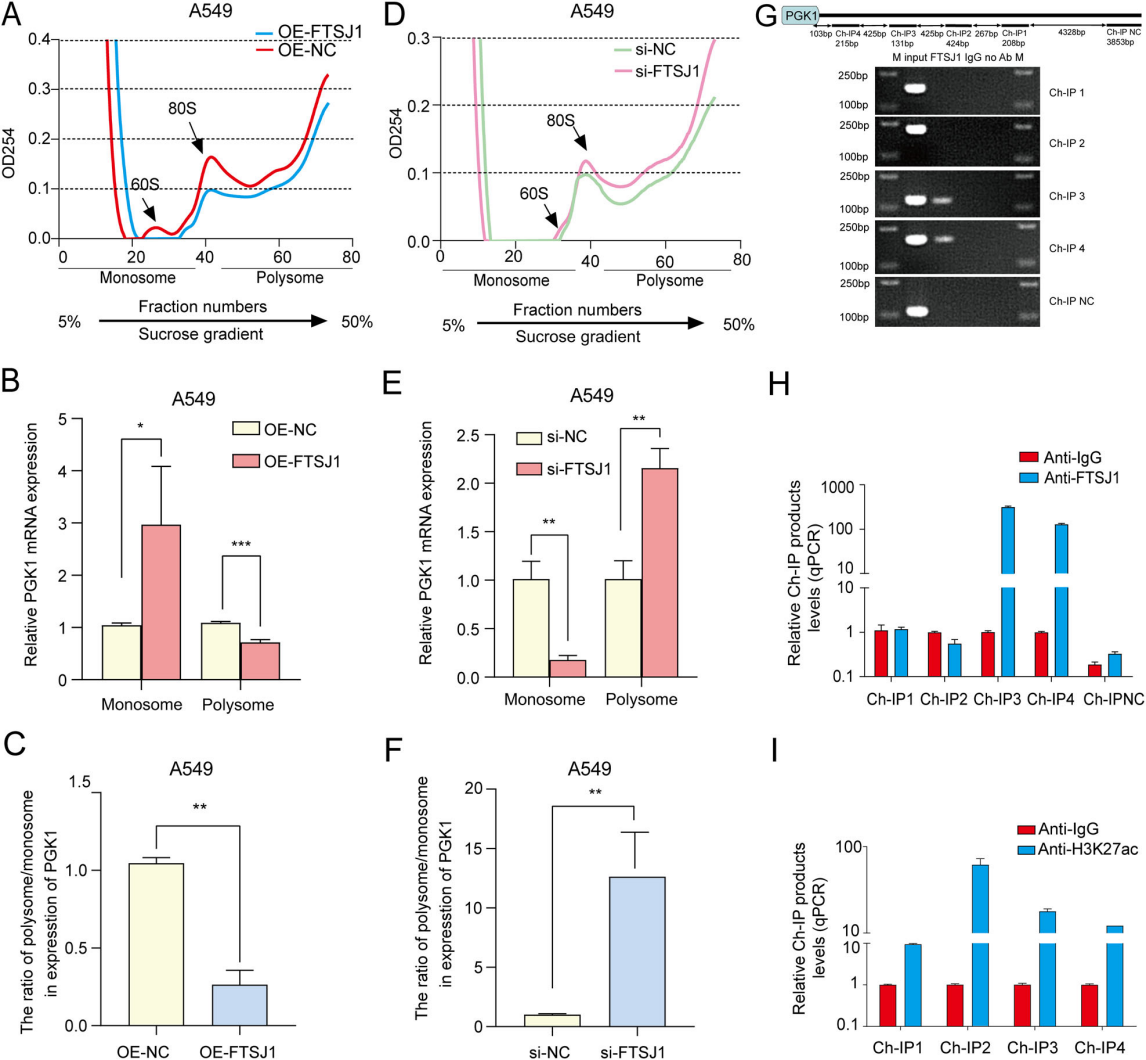
FTSJ1 regulates the translation and histone H3K27ac modification of PGK1 in NSCLC cells. **A** Polysome profiling shows a general translation suppression in FTSJ1-overexpression cells compared with that in NC cells. **B** Up-regulation of FTSJ1 decreases the abundance of PGK1 mRNA in the polysomes but increases the expression levels of PGK1 in monosomes in A549 cells. **C** Overexpression of FTSJ1 decreased the polysome to monosome ratio of PGK1 mRNA expression, indicating a reduction of PGK1 translation. **D** Polysome profiling of si-FTSJ1-treated A549 cells suggests a trend of increasing translation in comparison with control cells. **E** Inhibition of FTSJ1 increases the abundance of PGK1 mRNA in polysomes but decreases the expression levels of PGK1 in monosomes in A549 cells. **F** Downregulation of FTSJ1 enhances the polysome to monosome ratio of PGK1 mRNA expression, indicating an increase of PGK1 translation. **G** Schematic illustration of four potential binding sites (Ch-IP1, Ch-IP2, Ch-IP3, Ch-IP4) in the PGK1 promoter for FTSJ1 (top). ChIP assay indicates the binding of FTSJ1 and PGK1 (bottom). **H** ChIP-qPCR assay with anti-FTSJ1 antibody verifies the binding between FTSJ1 and two sites in the PGK1 promoter in A549 cells. **I** ChIP-qPCR assay shows that inhibition of FTSJ1 increases H3K27ac levels in PGK1 promoter region. Data are presented as mean ± SD, Student’s *t* test. *p < 0.05, **p < 0.01, ***p < 0.001. All *p* values are versus NC.

Given that FTSJ1 regulated the expressions of PGK1 protein, we hypothesized that FTSJ1 may target PGK1 through regulating its translation. To test this hypothesis, we conducted polysome fractionation experiments to separate the actively translating mRNAs that are associated with polysomes from the inactively translating mRNAs that are bounded with monosomes. Polysome profiles showed a decrease in polysome content in FTSJ1-overexpression cells (Fig. 6A), consistent with the observation that FTSJ1 induced a reduction in overall protein synthesis (Fig. 4F, G). Moreover, overexpression of FTSJ1 upregulated the expression of PGK1 mRNA in monosomes but downregulated the expression of PGK1 in polysomes, suggesting a decrease of PGK1 translation in A549 cells (Fig. 6B, C). On the contrary, downregulation of FTSJ1 increased the polysome content (Fig. 6D) and reduced the expression levels of PGK1 mRNA in monosomes but increased the abundance of PGK1 mRNA in polysomes (Fig. 6E, F), indicating an increase in translation initiation of PGK1. Collectively, these data suggest that FTSJ1 regulates PGK1 via modifying its translation in the ribosomes of NSCLC cells.

### FTSJ1 regulates H3K27ac abundance in the promoter region of PGK1

Histone modification alteration plays a vital role in epigenetic regulation of gene transcription. To explore whether PGK1 expression may be epigenetically regulated by FTSJ1, we used the UCSC genome browser (https://genome.ucsc.edu/) to analyze the genetic characteristics of PGK1 gene. The results showed that the PGK1 promoter region contains marks of H3K27ac, a histone modification for transcription activation. To examine whether FTSJ1 may combine to PGK1 promoter, we performed ChIP assays and found that two binding sites (−215 bp ~ −103bp, −647bp ~ −516bp) for FTSJ1 were located in the promoter region of PGK1 (Fig. 6G, H). Moreover, ChIP-qPCR assay revealed that knockdown of FTSJ1 significantly increased H3K27ac levels in PGK1 promoter region (Fig. 6I). These observations suggest that FTSJ1 may epigenetically modify PGK1 gene expression through increasing H3K27ac modification levels in PGK1 promoter.

### FTSJ1 expression levels are correlated with glycolysis metabolism in tumor tissues of NSCLC patients

**Fig. 7:**
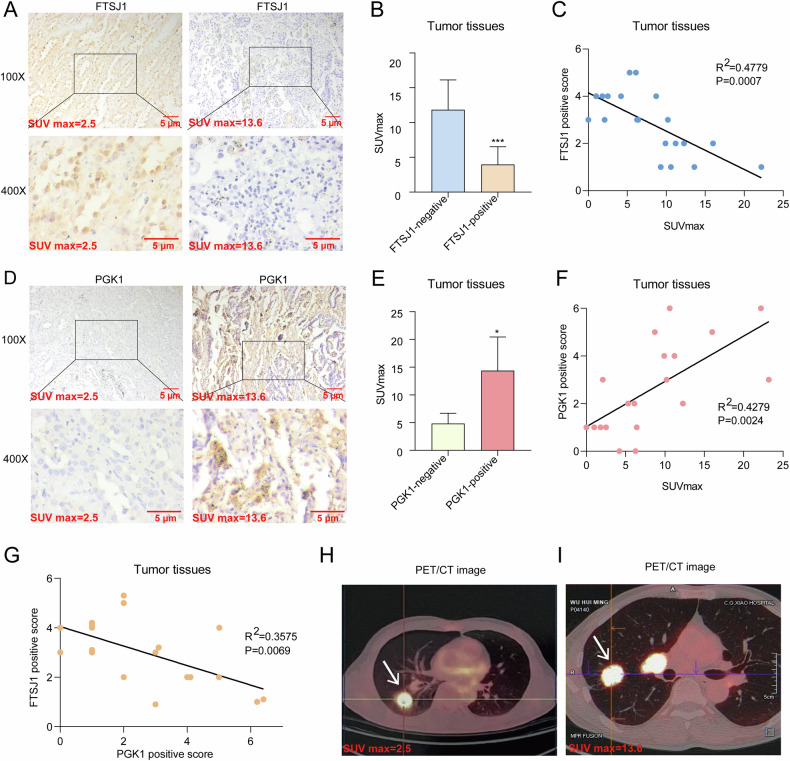
Association of FTSJ1 and PGK1 expression with glucose uptake in patients with NSCLC. **A** Representative immunohistochemical staining (IHC) images of high and low FTSJ1 expression in tumor tissues from NSCLC patients. Scale bar: 5 µm. **B** Mean SUVmax score in FTSJ1 negative NSCLC tissue samples was higher than that of positive tissues. **C** FTSJ1 expression level in tumor tissues was negatively correlated with SUVmax score. **D** Representative IHC images of high and low PGK1 expression in tumor tissues of NSCLC patients. Scale bar: 5 µm. **E** Mean SUVmax score in PGK1 positive tissues was higher than that of PGK1 negative samples. **F** PGK1 expression level in NSCLC tumor tissues was positively correlated with SUVmax score. **G** FTSJ1 expression level was negatively correlated with PGK1 expression in NSCLC tumor tissues. **H** Representative image of ^18^FDG PET-CT scan in NSCLC patients with high FTSJ1 expression. The arrow reveals lower glucose uptake. **I** Representative image of ^18^FDG PET-CT scan in NSCLC patients with low FTSJ1 expression level in tumor tissues. The arrow points to higher glucose uptake. Data are presented as mean ± SD. Two-tailed paired *t*-test. *p < 0.05, **p < 0.01, ***p < 0.001.

To assess the clinical significance of FTSJ1 in glucose metabolism in NSCLC, we investigated the relationship between SUVmax value (a surrogate indicant for aerobic glycolysis) of PET-CT scan and the expression levels of FTSJ1 and PGK1 proteins in tumor tissues resected from 19 NSCLC patients (Table S1). We found that tumors with higher FTSJ1 expression exhibited a decreased SUVmax value, while low FTSJ1 expression displayed increased SUVmax score (Fig. 7A, B). The expression levels of FTSJ1 in tumors were negatively correlated with the SUVmax values (Fig. 7C). In contrast, decreased PGK1 expression accompanied lower SUVmax score and higher score of PGK1 staining revealed high values of SUVmax (Fig. 7D, E). Integration of these two parameters showed that the IHC staining scores of PGK1 were positively correlated with SUVmax values (Fig. 7F). In consistent with the results from in vitro experiments, FTSJ1 protein expression levels in NSCLC tumor tissues were negatively correlated with that of PGK1 (Fig. 7G). Additionally, ^18^F-FDG PET/CT imaging showed that tumors with high FTSJ1 expression had a relatively weak SUVmax score (Fig. 7H), while tumors with lower FTSJ1 expression accompanied with higher SUVmax score. (Fig. 7I). Taken together, our data suggests that lower FTSJ1 expression level in tumor tissues reflects higher glycolysis metabolism in NSCLC.

## Discussion

While PM2.5 has been established as a risk factor for lung cancer incidence and mortality [4–6], the molecular mechanisms by which PM2.5 induces lung tumorigenesis remain incompletely understood. In this study, we demonstrated that FTSJ1, a tRNA methyltransferase, was downregulated by PM2.5 in vitro and in vivo. The decreased expression of FTSJ1 led to increased glycolysis metabolism in NSCLC cells. Further study confirmed that FTSJ1 expression level was negatively correlated with SUVmax score in patients with NSCLC. Mechanistically, low FTSJ1 expression promoted proliferation and glycolysis of NSCLC cells through upregulating the transcription and translation of glycolytic gene PGK1. Our work is the first to reveal the role of PM2.5-downregulated FTSJ1 in NSCLC and clarify its mechanisms.

Transfer RNAs (tRNAs) are fundamental biological molecules that decode genetic information from DNA into polypeptide during translation. To ensure tRNA stability, translational efficiency and fidelity, tRNAs are modified post-transcriptionally by different classes of tRNA-modifying enzyme genes [33]. The nucleosides in tRNA molecules may also be modified by environmental stresses, such as aging, starvation, and different stress conditions [23, 34]. For example, H_2_O_2_, MMS, and sodium arsenite (NaAsO_2_) could cause distinct tRNA modification change patterns that were linked to selective translation of codon-biased mRNAs [35, 36]. It has been documented that tRNA modifications are implicated in tumorigenesis of human cancers. For instance, deregulations of tRNA modification enzyme genes METTL1 and ALKBH1 induced impaired translation and aberrant cancer progression in vitro and in vivo [37, 38]. Thus, nucleoside modifications were not only manifested as a ‘signal’ for stress, but may also be used as a ‘regulatory module’ to timely adapt the cell to environmental changes [36]. Here, we found that PM2.5 suppressed the expression of tRNA modifying gene FTSJ1 in a dose-response fashion. Additionally, we showed that FTSJ1 contributed to NSCLC cell proliferation through interacting with glycolytic gene PGK1. These data suggest that FTSJ1 dysregulation may play a critical role in PM2.5-induced carcinogenesis.

Aerobic glycolysis, or Warburg effect, has been recognized as a central hallmark of human cancers, which involves elevated glucose uptake and lactate production even in the presence of abundant oxygen [39]. This phenomenon not only helps cancer cells to meet growth and proliferation demands, but also allows the use of intermediates of glycolysis for the production of nucleotides, amino acids, lipids, and NADH [40]. Growing evidence suggests that several chemical carcinogens (e.g. arsenic, heavy metals, and B[a]P) can regulate glycolytic reprogramming in carcinogenesis [41]. Whereas relatively little is known about the impact of PM2.5 on glycolytic reprogramming. Previously, we have found that PM2.5 could enhance glycolysis of NSCLC through upregulating DLAT translation and transcription [27]. But the molecular mechanisms underlying PM2.5-induced glycolysis reprogramming in NSCLC tumorigenesis remain unclear. Recently, emerging evidence has suggested that tRNA modifications might play important roles in the regulation of glycolysis metabolism in cancers. For example, the tRNA U34 enzyme genes promoted glycolysis in melanoma cells by regulating the translation of HIF-1α mRNA [42]. Decreased levels of Q-modification, catalyzed by eTGT at position 34 in the anticodon loop of tRNA, contributed to cancer cell survival and immune resistance through promoting glycolysis metabolism [43]. BCDIN3D methylated the modification of tRNA^His^, but BCDIN3D-depleted cells have increased levels of F1,6-BP, an inhibitor of glycolytic capacity [44]. Here, we showed that PM2.5 promoted NSCLC cell malignancy through a FTSJ1-mediated glycolysis reprogramming mechanism. Furthermore, we confirmed that the expression level of FTSJ1 was negatively correlated with SUVmax values of the PET-CT scan in patients with NSCLC. These findings expand our knowledge on the roles of tRNA modification in glycolysis reprogramming and uncover a novel mechanism of tRNA modifying gene in chemical-induced carcinogenesis.

Among the key glycolytic enzyme genes in glycolysis pathway, PGK1 was the most significantly dysregulated one by FTSJ1. Downregulation of FTSJ1 led to increased expression of PGK1 at both the mRNA and protein levels. In addition, ribosome profiling and qRT-PCR assays indicated that knockdown of FTSJ1 resulted in upregulation of PGK1 mRNA translation in NSCLC cells, highlighting the importance of FTSJ1-PGK1 regulatory axis in the pathogenesis of NSCLC. PGK1 (Phosphoglycerate kinase 1) and pyruvate kinase M2 (PKM2) are the only two enzymes that control ATP production during aerobic glycolysis in cells [45]. But the enzyme activity of PKM2 in cancer cells is very low, thus PGK1 plays a more important role in regulating glycolysis metabolism in cancer cells [46]. PGK1 catalyzes the conversion of 1,3-diphosphoglycerate (1,3-BPG) to 3-phosphoglycerate (3-PG) and produces one molecule of ATP [47]. In addition to cell metabolism regulation, PGK1 is involved in multiple biological activities, including angiogenesis, autophagy and DNA repair [48]. PGK1 expression is upregulated in many types of human cancer [49]. PGK1 is also associated with chemoradiotherapy resistance and poor prognosis of cancer patients [50]. As a major member of HIF-1α signaling pathway, it has been proved that PGK1 is directly regulated by HIF-1α in many cancers [51]. But there is no report on the relationship between PGK1 and any tRNA modifying gene. Here, we demonstrated, for the first time, that PGK1 was transcriptionally and translationally regulated by FTSJ1 in NSCLC cells. Importantly, rescue experiments confirmed that FTSJ1 contributed to proliferation and glycolysis of NSCLC cells in a PGK1-dependent fashion. These results highlight the significance of FTSJ1 in PGK1-mediated glycolysis in PM2.5-associated NSCLC.

It is noteworthy that PM2.5 has been recognized as a major risk factor for lung cancer in never smokers (LCNS) [52, 53], which is mostly diagnosed as adenocarcinoma (LUAD) subtype and occurs more frequently in Asian and Hispanic young women with advanced disease [54]. LCNS also has higher mutation prevalence on EGFR, ALK, and ROS1 but lower mutation rate on KRAS [54, 55]. Previously, it has been observed that there were significant genomic differences between LCNS and smoked LC patients [56]. To clarify the oncogenic mechanisms of PM2.5 in NSCLC, it would be interesting to investigate whether PM2.5 may have any effect on EGFR, ALK, or KRAS mutations in future studies.

Our study has several limitations. First, we focused on the role of FTSJ1 in glycolysis metabolism in NSCLC cells. However, it is known that many cancer cells display enhanced glycolysis and suppressed mitochondrial tricarboxylic acid (TCA) cycle [57]. Whether FTSJ1 may have any effect on TCA cycle needs to be further studied. Second, although we found many FTSJ1-dysregulated genes in NSCLC cells, only PGK1 was uncovered. It is possible that other FTSJ1-modified genes could also play a role in FTSJ1-mediated mechanisms. Third, our findings from a relatively small sample size highlighted that FTSJ1 may serve as a potential glycolytic biomarker for NSCLC. More clinical research in larger sample size is warranted to confirm the results of the present study.

In summary, our findings demonstrate that downregulation of FTSJ1 by PM2.5 promotes NSCLC malignancy via upregulating PGK1-mediated glycolysis reprogramming in NSCLC cells. Targeted upregulation of FTSJ1 expression may be a promising therapeutic strategy against NSCLC.

## Supplementary information

Supplementary materials

Supplementary materials (Western blot images)

## Data Availability

The data supporting the findings of this study are available from the corresponding author upon reasonable request.
