# Supplementary material for: Downregulation of tRNA methyltransferase FTSJ1 by PM2.5 promotes glycolysis and malignancy of NSCLC via facilitating PGK1 expression and translation
Source: Cell Death Dis. 2024 Dec 18;15(12):911. doi: 10.1038/s41419-024-07287-0 (PMC11655989; doi:10.1038/s41419-024-07287-0)
Supplement: Supplementary file 1 — Supplementary materials [file 41419_2024_7287_MOESM1_ESM.pdf]

# Supplementary materials

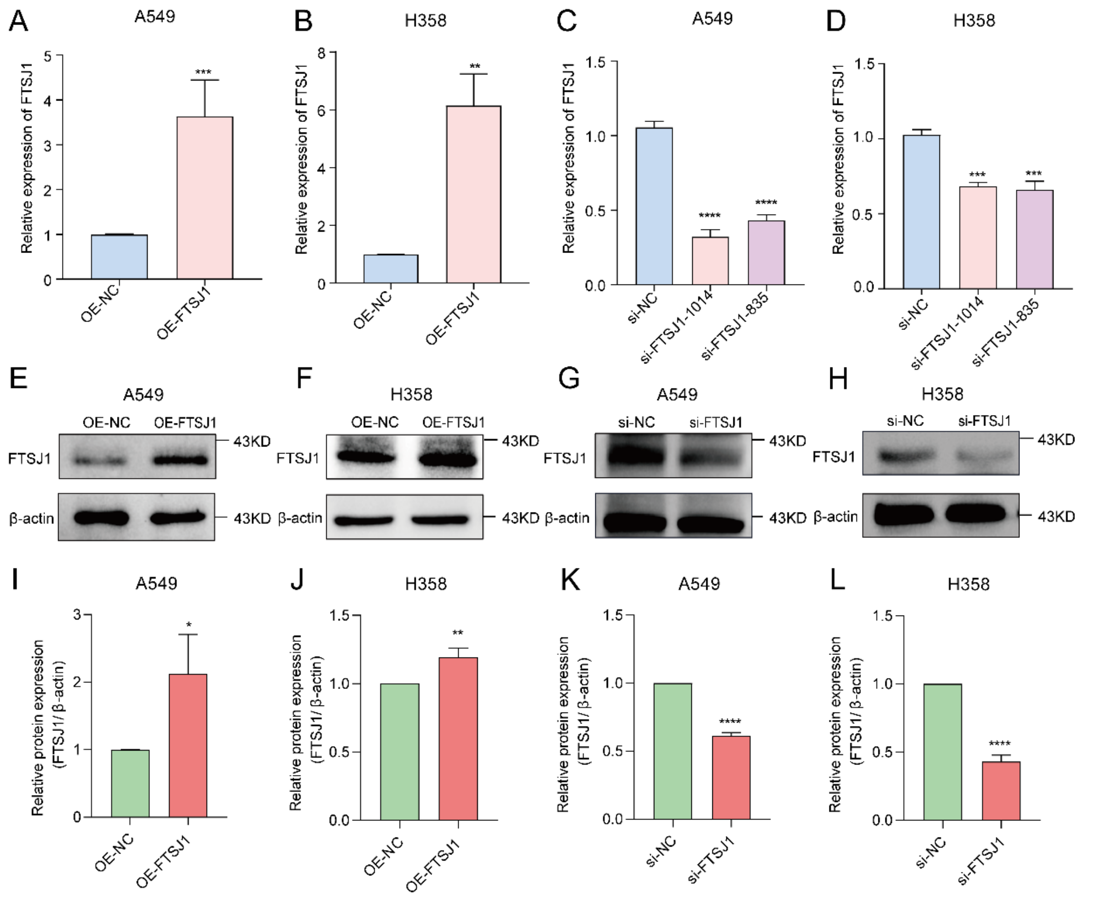


**Fig. S1 Transfection efficiency of FTSJ1 and si-FTSJ1 in NSCLC cells.** (**A**) Transfection efficiency of FTSJ1-overexpression vector in A549 cells. (**B**) Transfection efficiency of FTSJ1- overexpression vector in H358 cells. (**C**) Transfection efficiency of si-FTSJ1- in A549 cells. (**D**) Transfection efficiency of si-FTSJ1 in H358 cells. (**E, I**) Transfection efficiency of OE-FTSJ1 at protein level in A549 cells. (**F, J**) Transfection efficiency of OE-FTSJ1 at protein level in H358 cells. (**G, K**) Transfection efficiency of si-FTSJ1 at protein level in A549 cells. (**H, L**) Transfection efficiency of si-FTSJ1 at protein level in H358 cells. * *P<0.05; ** P<0.01; *** P<0.001*. All P values are versus NC.

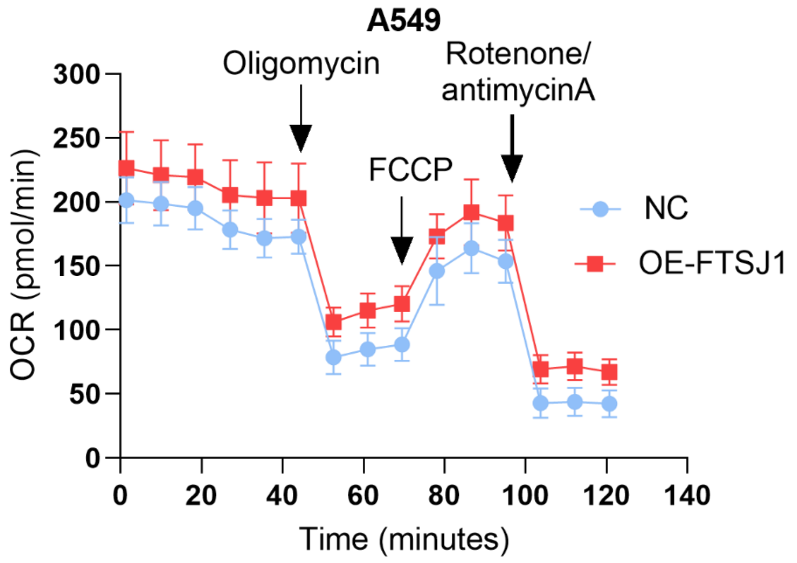


**Fig. S2 Effect of FTSJ1 overexpression on oxygen consumption rate (OCR) in NSCLC cells.** Up-regulation of FTSJ1 promoted OCR level of A549 cells.

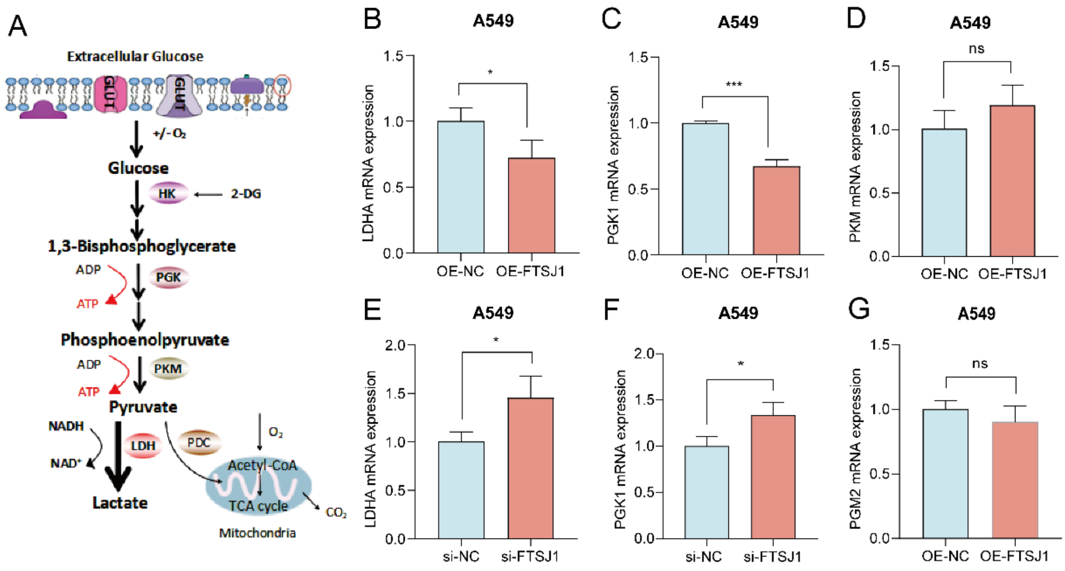


**Fig. S3 Impact of FTSJ1 on the expression level of key glycolytic genes.**

(A) Schematic diagram of glycolysis pathway. (B) Overexpression of FTSJ1 suppresses LDHA gene expression in A549 cells. (C) Upregulation of FTSJ1 inhibits PGK1 gene expression in A549 cells. (D) Overexpression of FTSJ1 has limited effect on PKM gene expression in A549 cells. (E) Knockdown of FTSJ1 promotes LDHA expression in A549 cells. (F) Downregulation of FTSJ1 enhances PGK1 expression in A549 cells. (G) Silencing of FTSJ1 has limited effect on PGM2 expression. *$P < 0.05$, **$P < 0.01$, ***$P < 0.001$. All P values are versus NC.

**Table S1. Characteristics of patients with NSCLC recruited for PET-CT scan and IHC staining**

| ID | Sex | Age at diagnosis | TNM Stage | Stage | SUVmax value (PET/CT) |
|---|---|---|---|---|---|
| Case 1 | Male | 66 | pT2aN0M0 | IB | 2.5 |
| Case 2 | Male | 64 | pT2aN1M0 | IIB | 13.6 |
| Case 3 | Male | 52 | pT2aN1M0 | IIA | 12.3 |
| Case 4 | Male | 64 | pT2aN0M0 | IB | 6.3 |
| Case 5 | Male | 72 | pT2aN3M1a | IV | 6.4 |
| Case 6 | Male | 66 | pT2aN2M0 | IIIA | 9.9 |
| Case 7 | Male | 73 | pT2aN0M0 | IB | 22.2 |
| Case 8 | Male | 69 | pT1cN0M0 | IA3 | 4.2 |
| Case 9 | Male | 55 | pT4N0M0 | IIIA | 11.2 |
| Case 10 | Female | 71 | pT2aN0M0 | IB | 16.0 |

| | | | | | |
|---|---|---|---|---|---|
| Case 11 | Female | 64 | pT2aN0M0 | IB | 1.8 |
| Case 12 | Male | 61 | pT2bN0M0 | IIA | 6.1 |
| Case 13 | Male | 79 | pT1cN0M0 | AI3 | 8.7 |
| Case 14 | Female | 71 | pT1bN0M0 | IA2 | 0 |
| Case 15 | Female | 39 | pT2aN0M0 | IB | 2.1 |
| Case 16 | Female | 67 | pT1bN2M0 | IIIA | 5.3 |
| Case 17 | Female | 76 | pT3N0M0 | IIB | 10.2 |
| Case 18 | Female | 56 | T1aN0M0 | IA3 | 1.0 |
| Case 19 | Male | 63 | pT1cNxM1a | IVA | 10.6 |

**Table S2**. **Primers, oligos, and antibodies used in this study**

| Oligo names | Sequences (5΄-3΄) | Source |
|---|---|---|
| FTSJ1-qPCR-F | CCATTCTTACGACCCAGATTTCA | Guangzhou RiboBio |
| FTSJ1-qPCR-R | CCCTCTAGGTCCAGTGGGTAAC | Guangzhou RiboBio |
| GAPDH-qPCR-F | GAACGGGAAGCTCACTGG | Guangzhou RiboBio |
| GAPDH-qPCR-R | GCCTGCTTCACCACCTTCT | Guangzhou RiboBio |
| ACTB-qPCR-F | TCAAGATCATTGCTCCTCCTGAG | Guangzhou RiboBio |
| ACTB-qPCR-R | ACATCTGCTGGAAGGTGGACA | Guangzhou RiboBio |
| FTSJ1-si-1014sense | GCAGCCGGAACUCUAGCAUTT | Suzhou GenePharma |
| FTSJ1-si-1014antisense | AUGCUAGAGUUCCGGCUGCTT | Suzhou GenePharma |
| FTSJ1-si-835 sense（5'-3'） | CCAUGAUGUUGAUGAGUAUTT | Suzhou GenePharma |
| FTSJ1-si-835antisens（5'-3'） | AUACUCAUCAACAUCAUGGTT | Suzhou GenePharma |

| | | |
|---|---|---|
| PGK1- qPCR-F | CCAGAGGATTAAGGCTGCTGT | Guangzhou RiboBio |
| PGK1- qPCR-R | GTGGCTCATAAGGACTACCGAC | Guangzhou RiboBio |
| Si-NC | Ribobio, # siN0000001-1 | Guangzhou RiboBio |
| PGK1 ChIP 1F | 5'TTGCTCAGGCCTGCTCTCAC 3' | Guangzhou RiboBio |
| PGK1 ChIP 1R | 5'TCAATTTTGTCTCAGAGTTTAC AC 3' | Guangzhou RiboBio |
| PGK1 ChIP 2F | 5'CAGGAAGAACTCGCAAGAAC 3' | Guangzhou RiboBio |
| PGK1 ChIP 2R | 5'CGCTGCCTAGACAGAGCTGA 3' | Guangzhou RiboBio |
| PGK1 ChIP 3F | 5'CATCAATCATGCCATAACCAG 3' | Guangzhou RiboBio |
| PGK1 ChIP 3R | 5'TTCTGCTTCTTAAGGCTTACT 3' | Guangzhou RiboBio |
| PGK1 ChIP 4F | 5'TCACGTCCGTTCGCAGCGTC 3' | Guangzhou RiboBio |
| PGK1 ChIP 4R | 5'ATTGGCCACAGCCCATCGCG 3' | Guangzhou RiboBio |

| | | |
|---|---|---|
| PGK1 ChIP-NCF | 5'TGTAGGTTCACTGGGGGCAC 3' | Guangzhou RiboBio |
| PGK1 ChIP-NCR | 5'TCATCCATAAGCTACATTAGG 3' | Guangzhou RiboBio |
| **Antibodies** | **Source** | |
| FTSJ1 antibody for Western blot | Abcam (ab227259) | |
| β -actin antibody for Western blot | CST (4970) | |
| PGK1 antibody for Western blot | Proteintech (17811-1-AP) | |
| FTSJ1 antibody for IHC | Atlas (HPA002718) | |
| PGK1 antibody for IHC | Proteintech (17811-1-AP) | |
| Peroxidase-Conjugated Goat anti-Rabbit IgG (H+L) | ZSGB-BIO (ZB-5301) | |
| Anti- H3K27ac for ChIP | Abcam (Ab4729) | |
| Anti-FTSJ1 for ChIP | SANTA CRUZ (SC-390355) | |
