# Supplementary figures and images for: Downregulation of tRNA methyltransferase FTSJ1 by PM2.5 promotes glycolysis and malignancy of NSCLC via facilitating PGK1 expression and translation
Source: Cell Death Dis. 2024 Dec 18;15(12):911. doi: 10.1038/s41419-024-07287-0 (PMC11655989; doi:10.1038/s41419-024-07287-0)

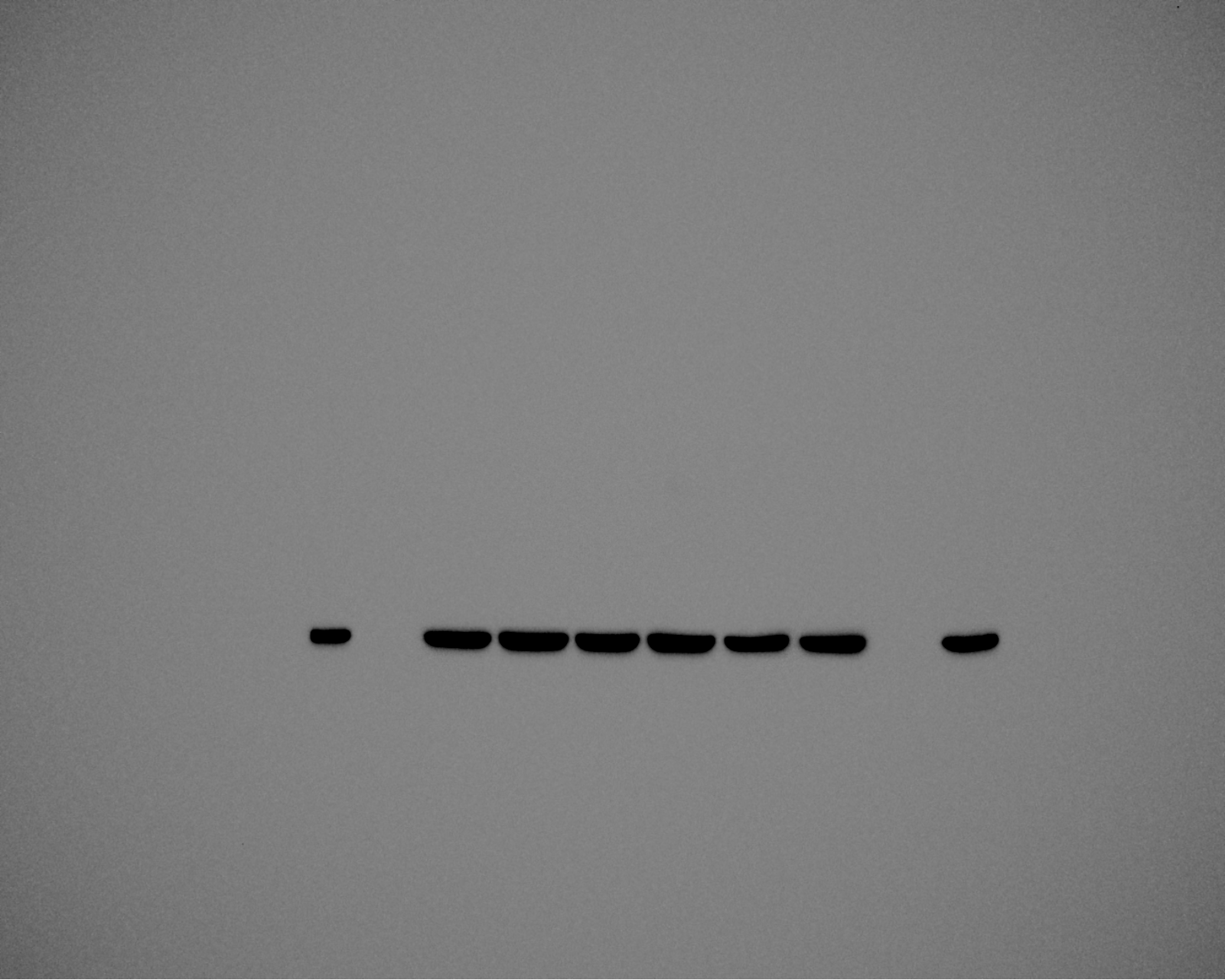

Supplement: Supplementary file 2 — Supplementary materials (Western blot images) [file 41419_2024_7287_MOESM2_ESM.zip › Suppl. materials_WB images/a549 0~400 actb_4(Chemiluminescence).tif]

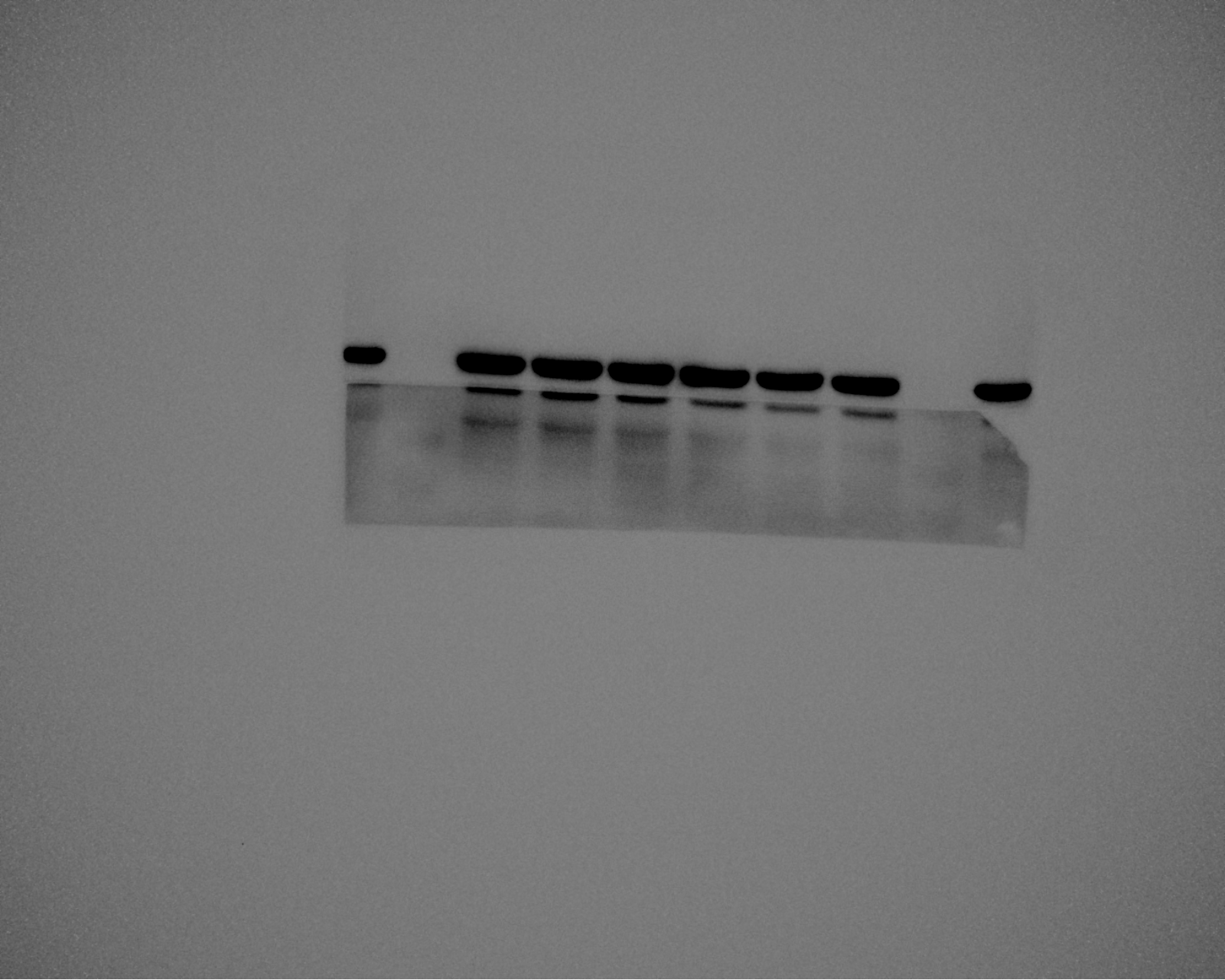

Supplement: Supplementary file 2 — Supplementary materials (Western blot images) [file 41419_2024_7287_MOESM2_ESM.zip › Suppl. materials_WB images/a549 ftsj1 &actb _2(Chemiluminescence).tif]

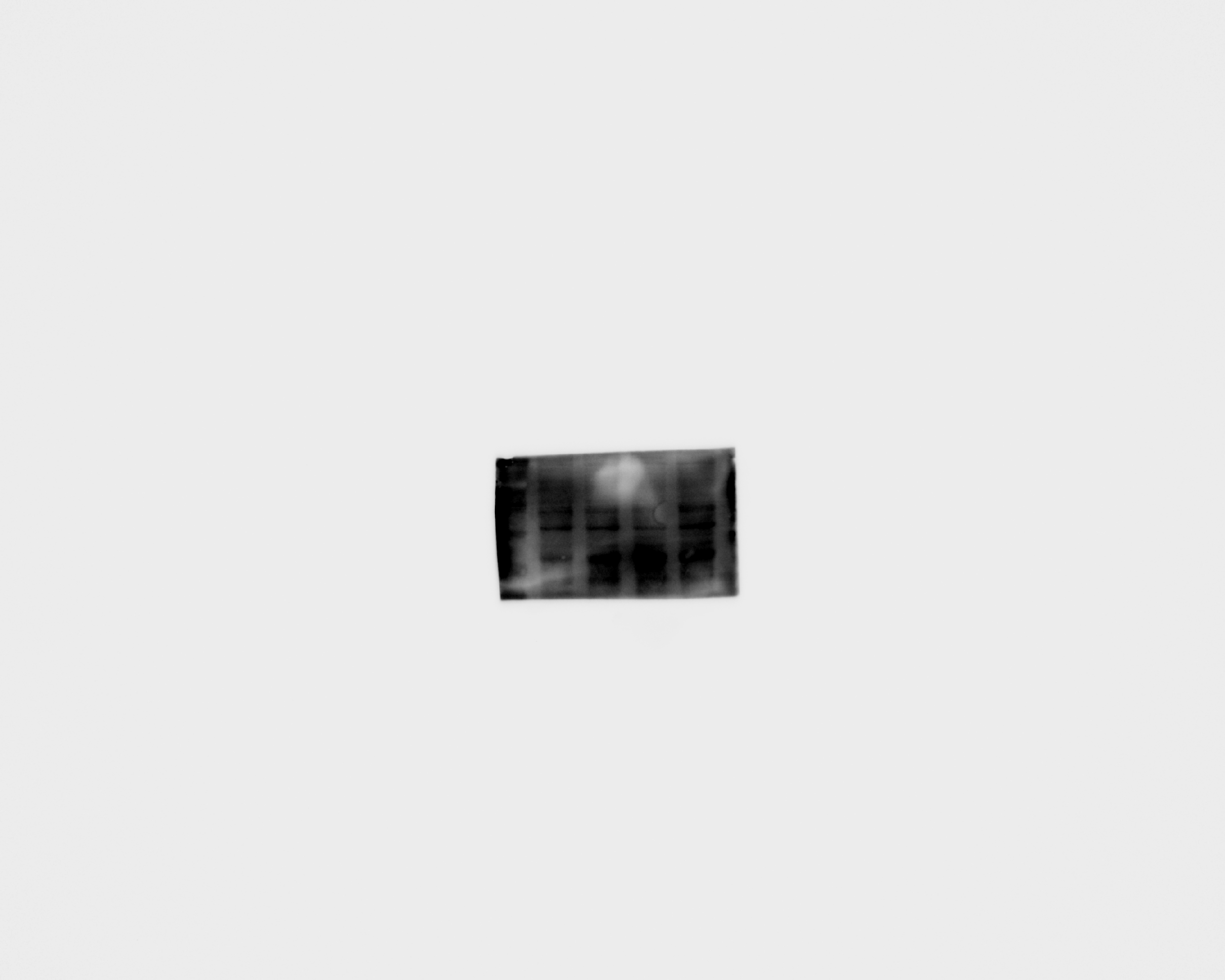

Supplement: Supplementary file 2 — Supplementary materials (Western blot images) [file 41419_2024_7287_MOESM2_ESM.zip › Suppl. materials_WB images/a549 oeFTSJ1 actb_2(Chemiluminescence).tif]

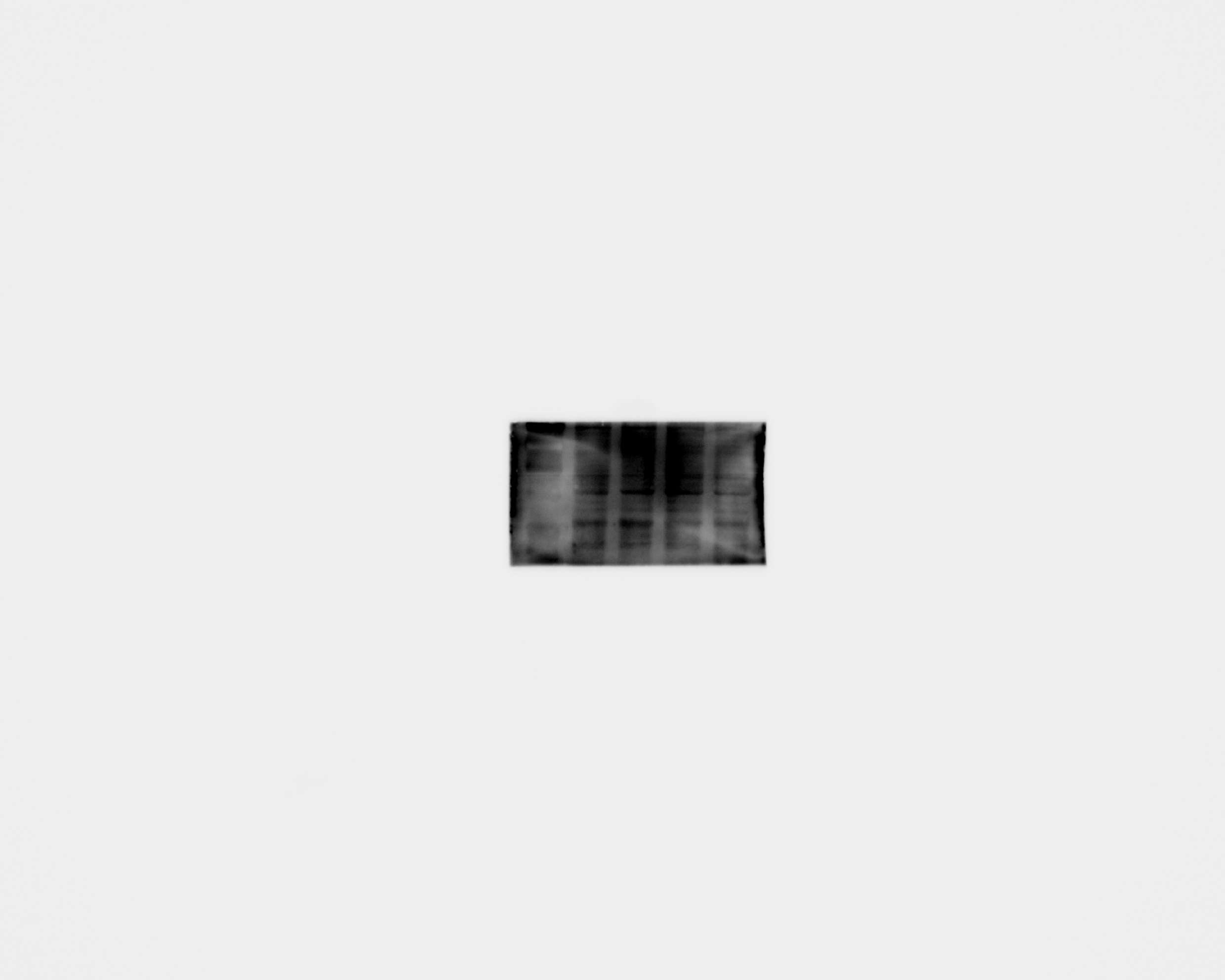

Supplement: Supplementary file 2 — Supplementary materials (Western blot images) [file 41419_2024_7287_MOESM2_ESM.zip › Suppl. materials_WB images/A549 OEFTSJ1 pgk1_2(Chemiluminescence).tif]

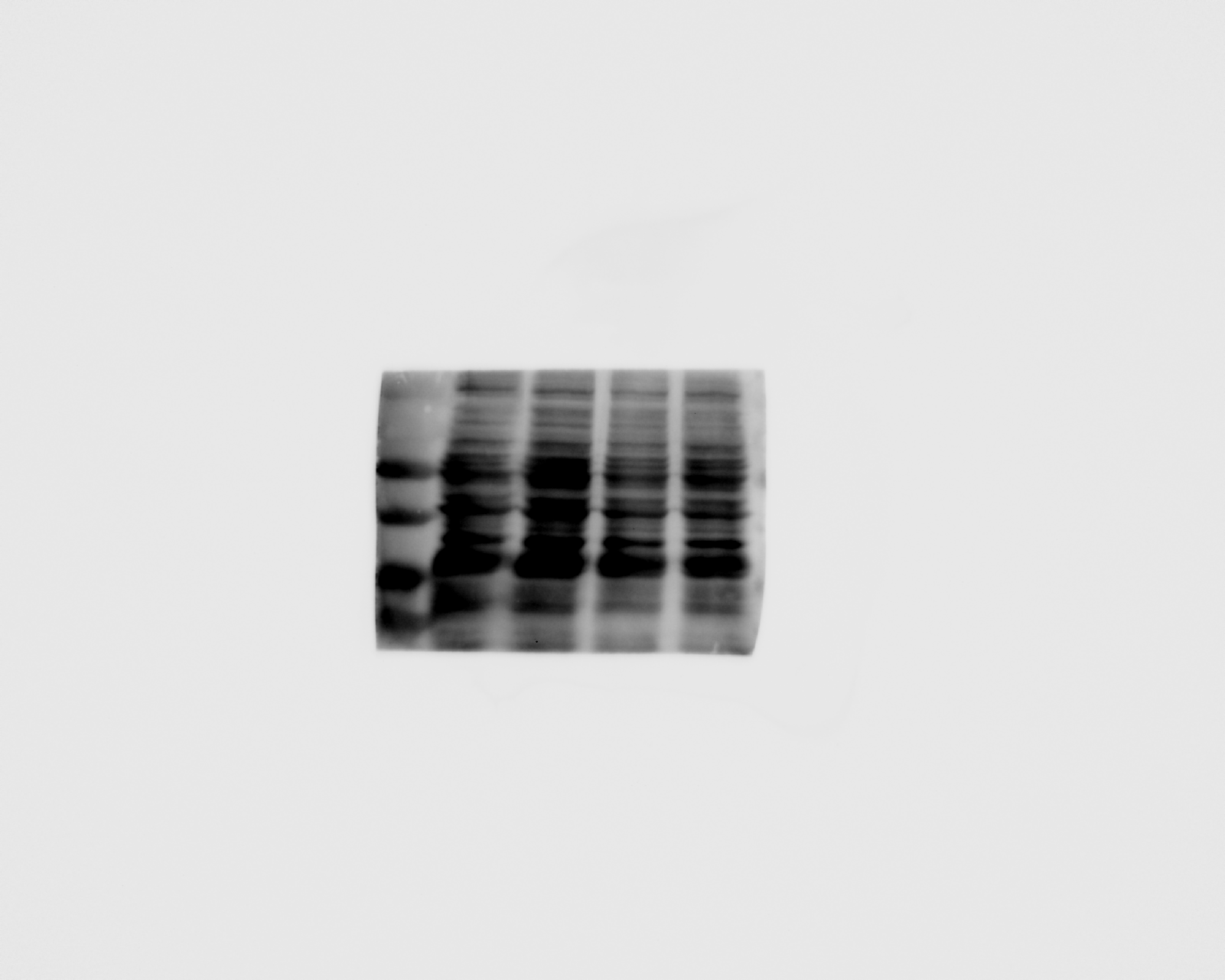

Supplement: Supplementary file 2 — Supplementary materials (Western blot images) [file 41419_2024_7287_MOESM2_ESM.zip › Suppl. materials_WB images/a549 si-ftsj1 actb_1(Chemiluminescence).tif]

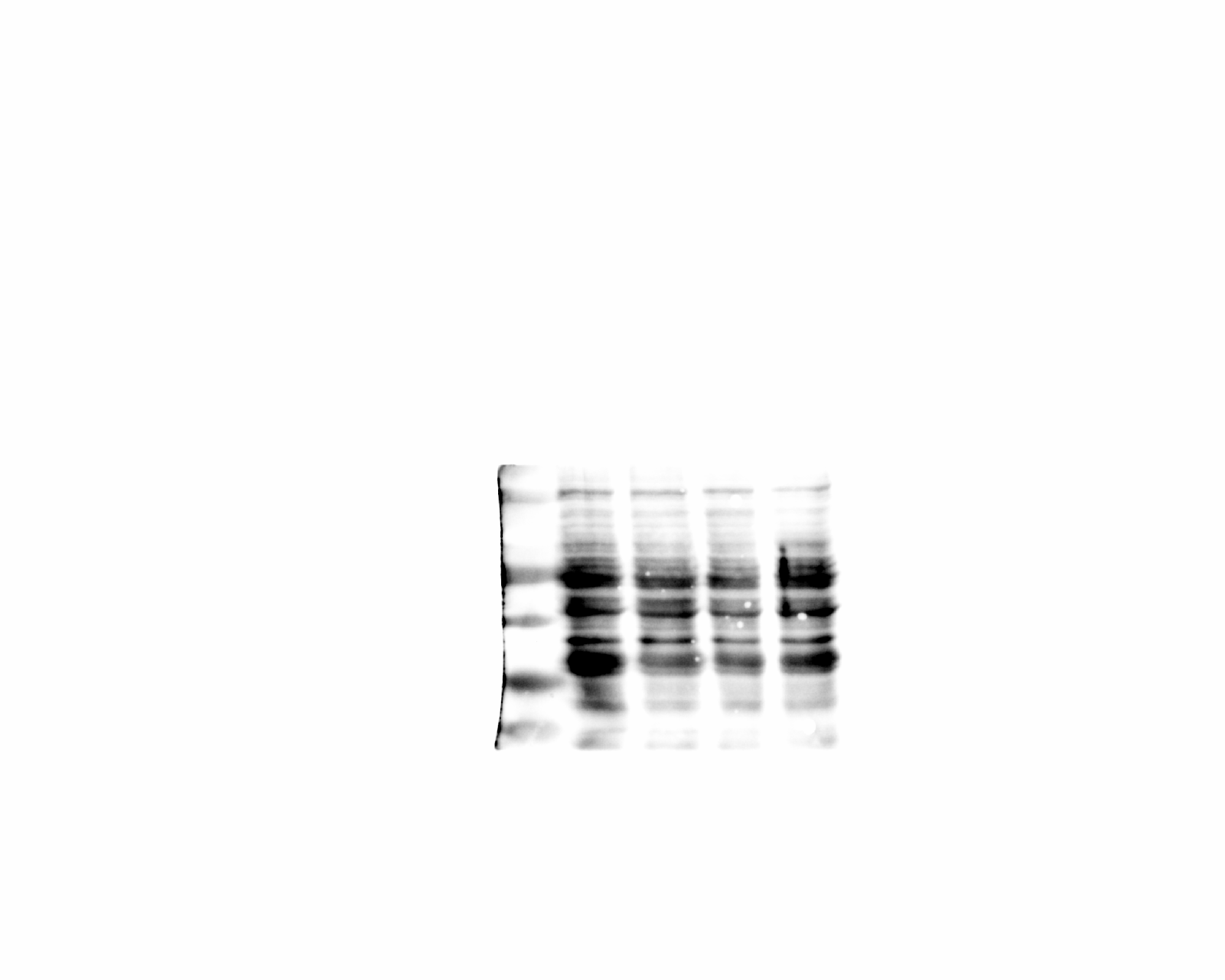

Supplement: Supplementary file 2 — Supplementary materials (Western blot images) [file 41419_2024_7287_MOESM2_ESM.zip › Suppl. materials_WB images/a549 si-ftsj1 PGK1_1(Chemiluminescence).tif]

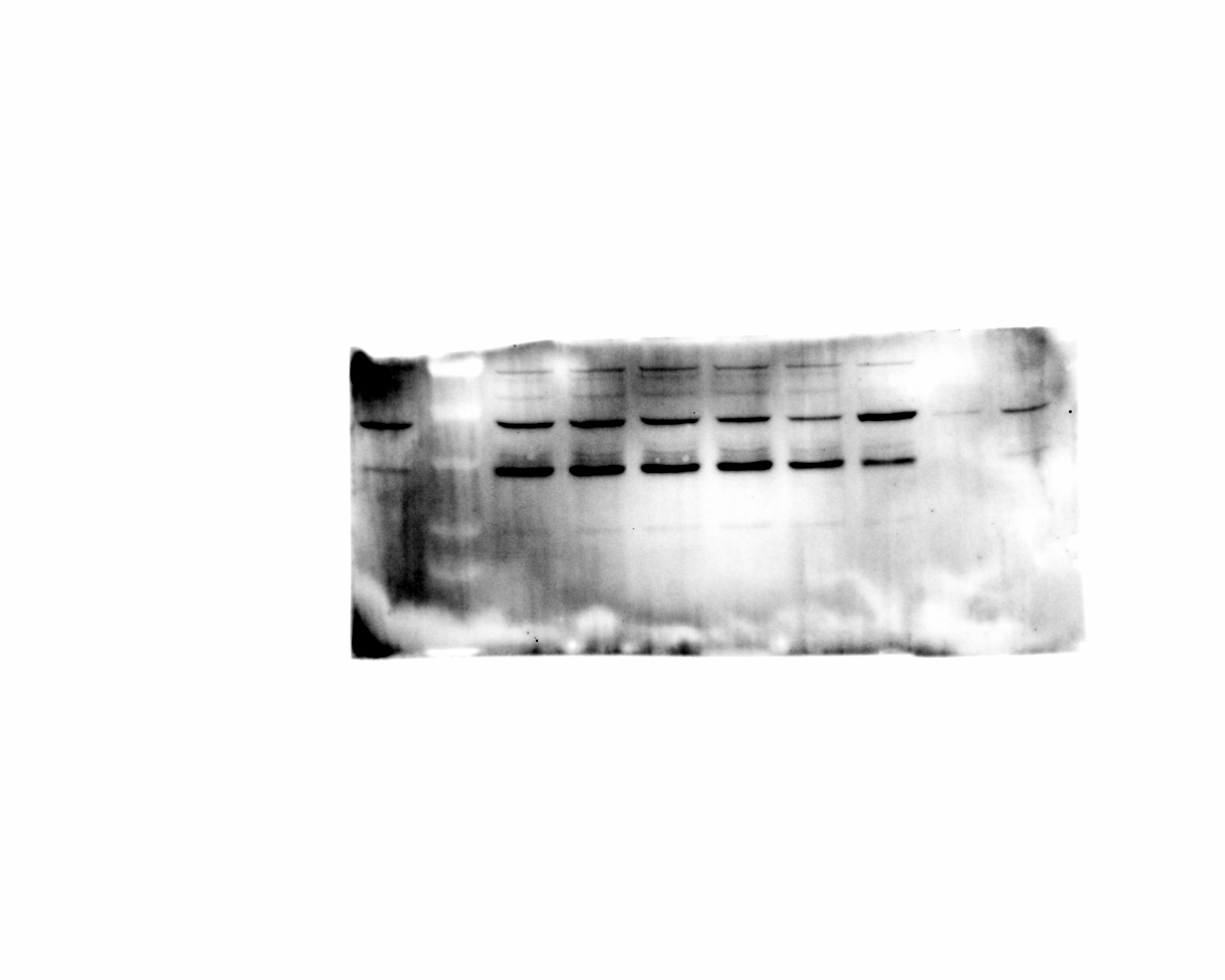

Supplement: Supplementary file 2 — Supplementary materials (Western blot images) [file 41419_2024_7287_MOESM2_ESM.zip › Suppl. materials_WB images/BEAS-2B 0~400 ftsj1 -2(Chemiluminescence).tif]

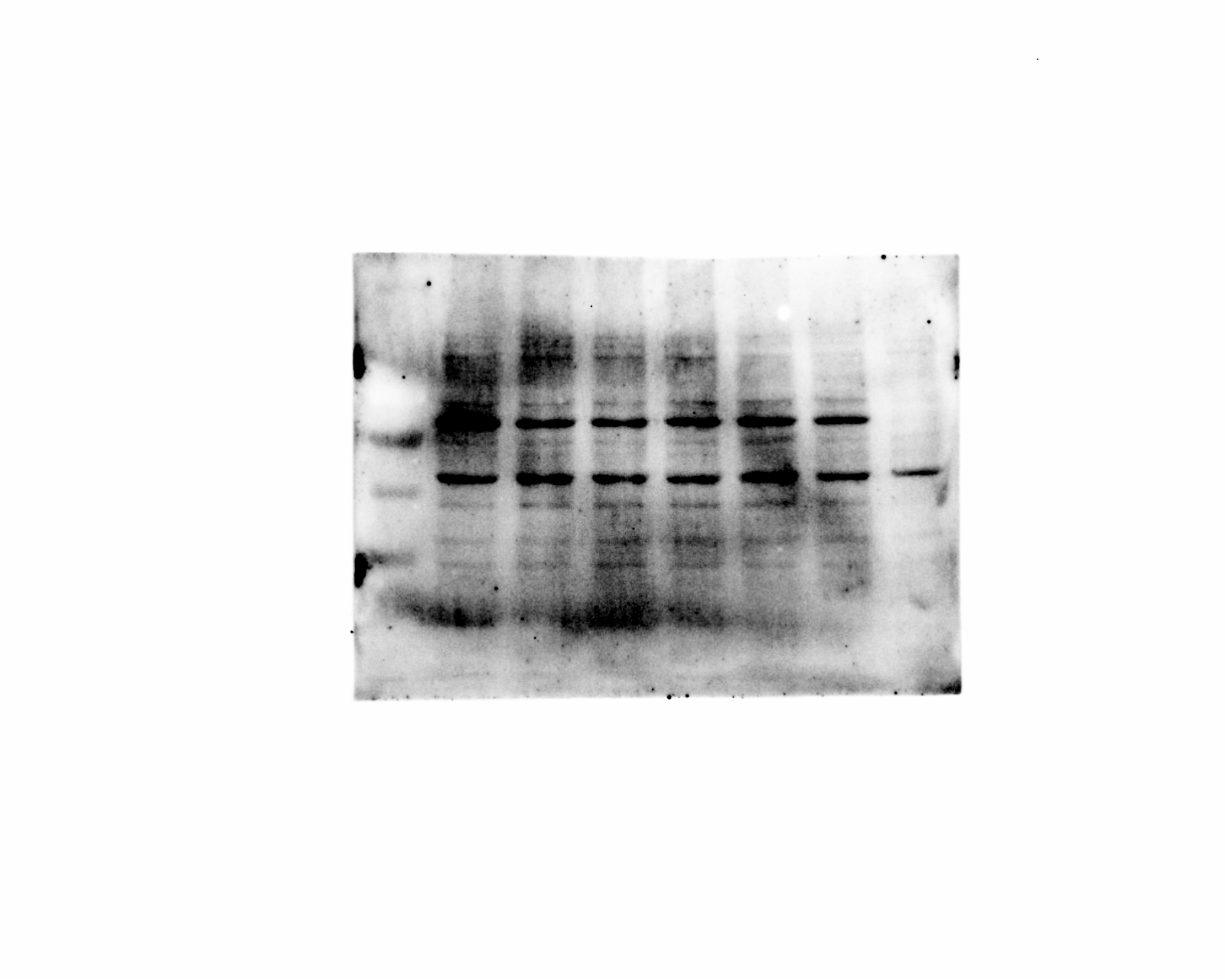

Supplement: Supplementary file 2 — Supplementary materials (Western blot images) [file 41419_2024_7287_MOESM2_ESM.zip › Suppl. materials_WB images/BEAS-2B actb 0~400(Chemiluminescence).tif]

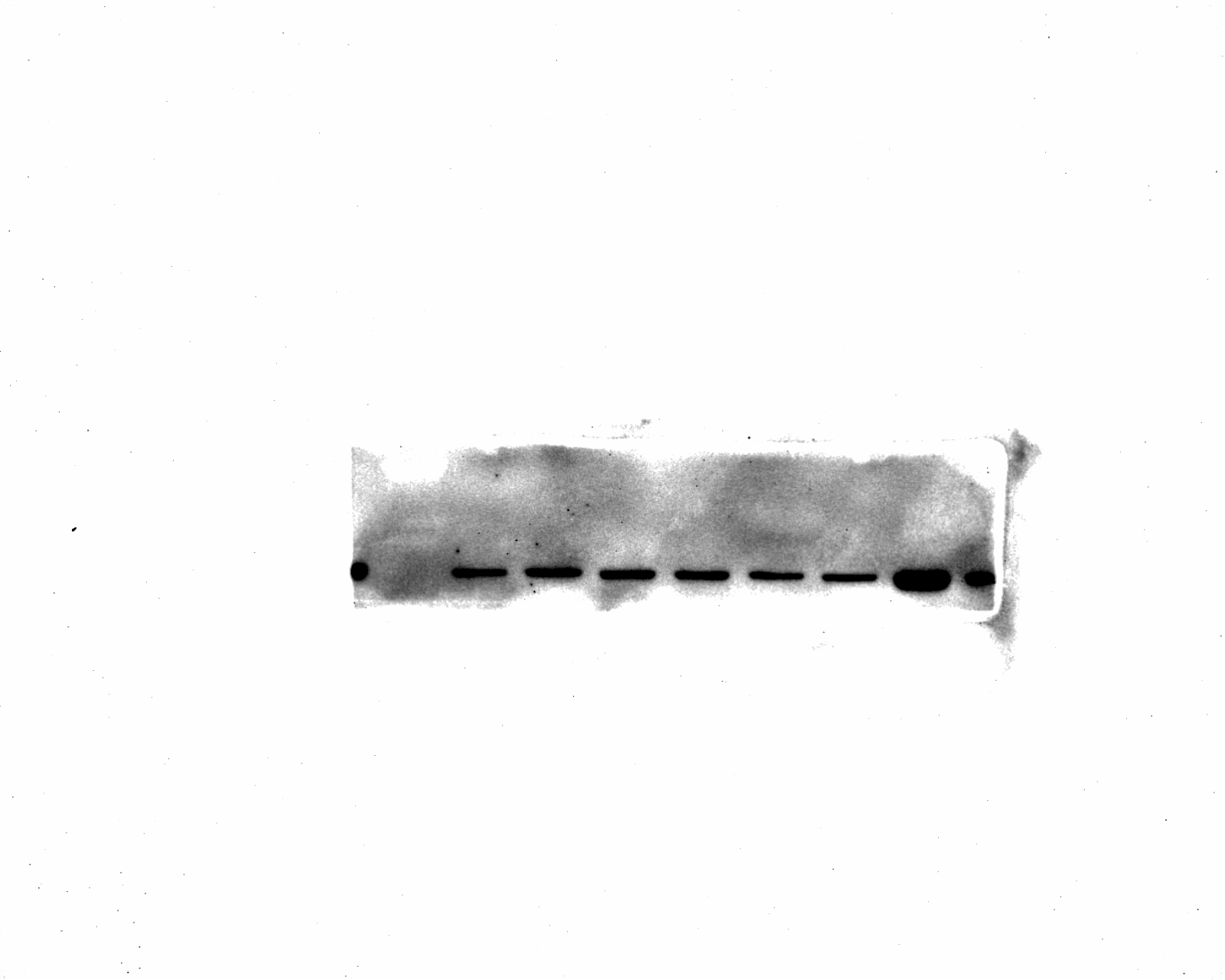

Supplement: Supplementary file 2 — Supplementary materials (Western blot images) [file 41419_2024_7287_MOESM2_ESM.zip › Suppl. materials_WB images/h358 actb0~400_3(Chemiluminescence).tif]

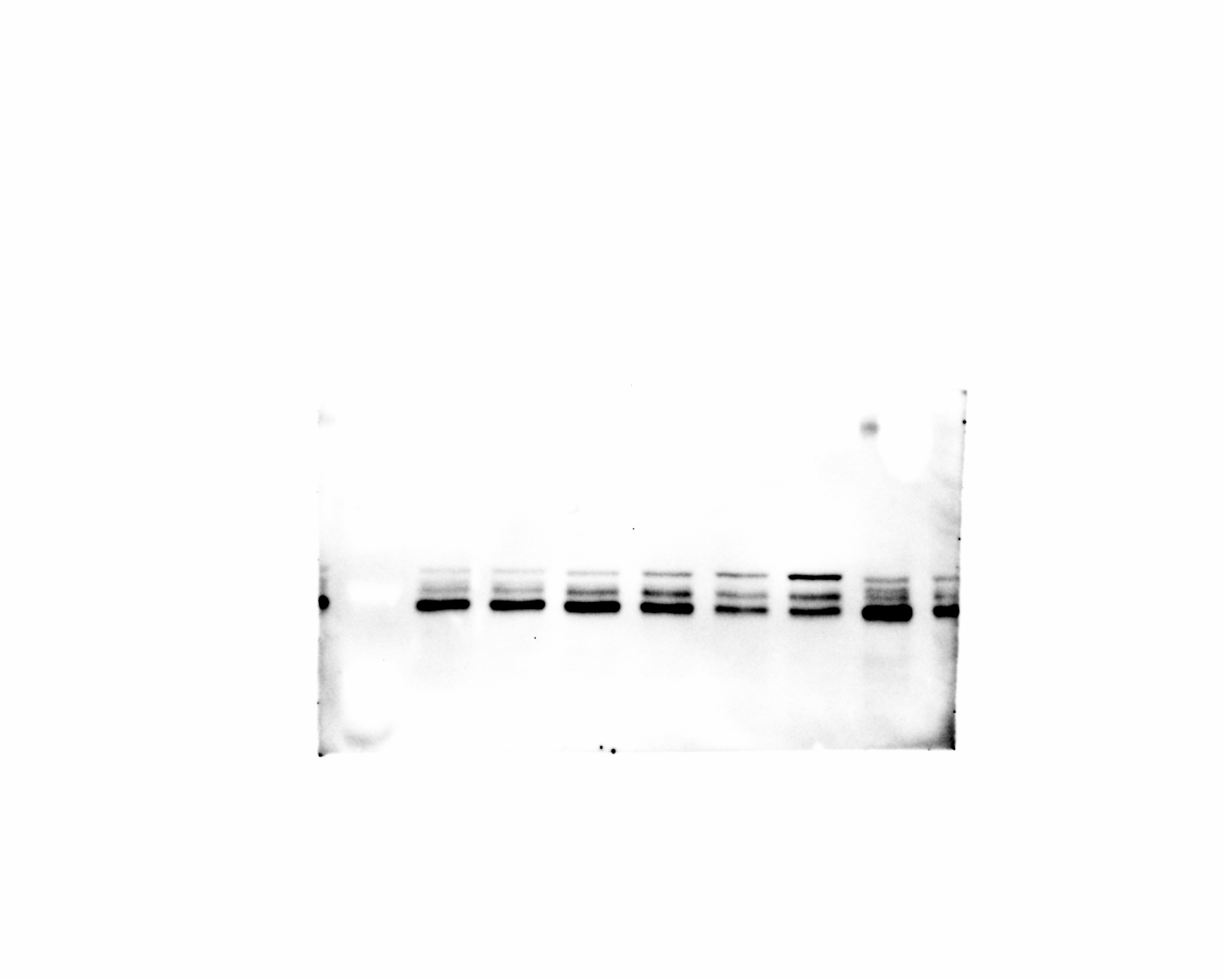

Supplement: Supplementary file 2 — Supplementary materials (Western blot images) [file 41419_2024_7287_MOESM2_ESM.zip › Suppl. materials_WB images/h358 ftsj1 0~400 1229_1(Chemiluminescence).tif]

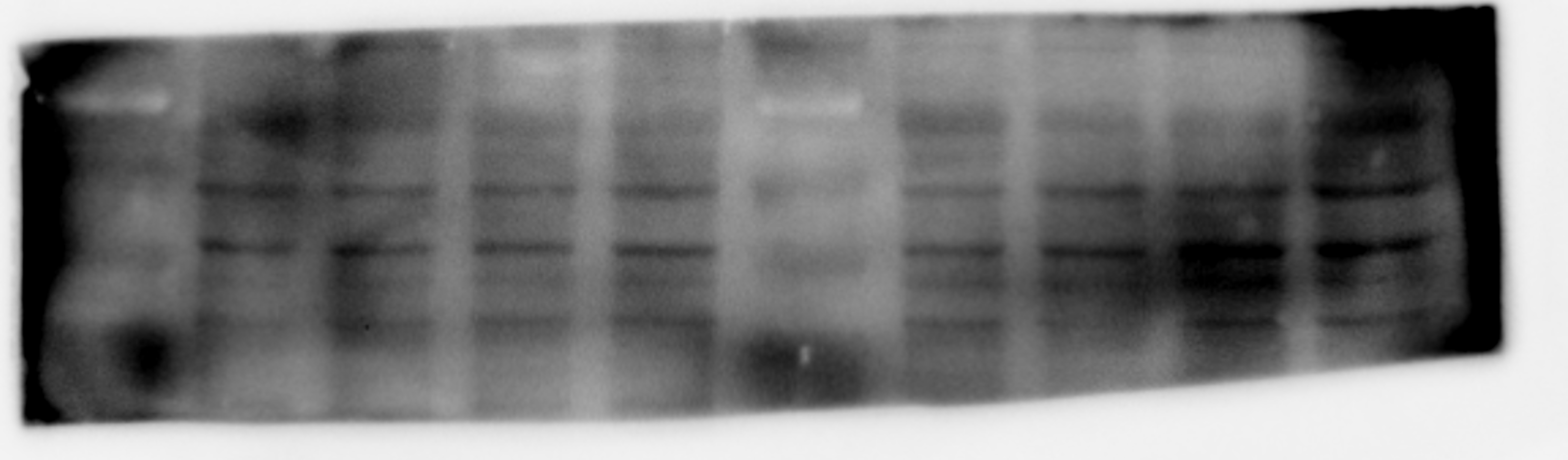

Supplement: Supplementary file 2 — Supplementary materials (Western blot images) [file 41419_2024_7287_MOESM2_ESM.zip › Suppl. materials_WB images/H358 OEFTSJ1 ACTB.tif]

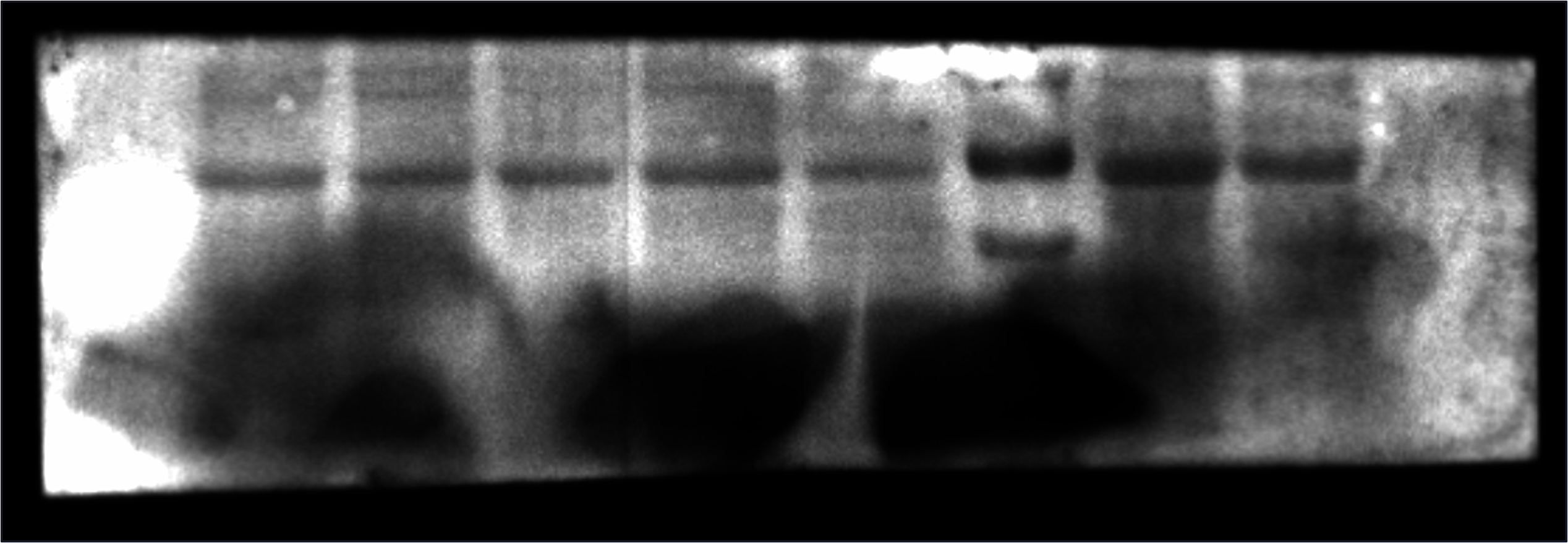

Supplement: Supplementary file 2 — Supplementary materials (Western blot images) [file 41419_2024_7287_MOESM2_ESM.zip › Suppl. materials_WB images/H358 OEFTSJ1 PGK1.tif]

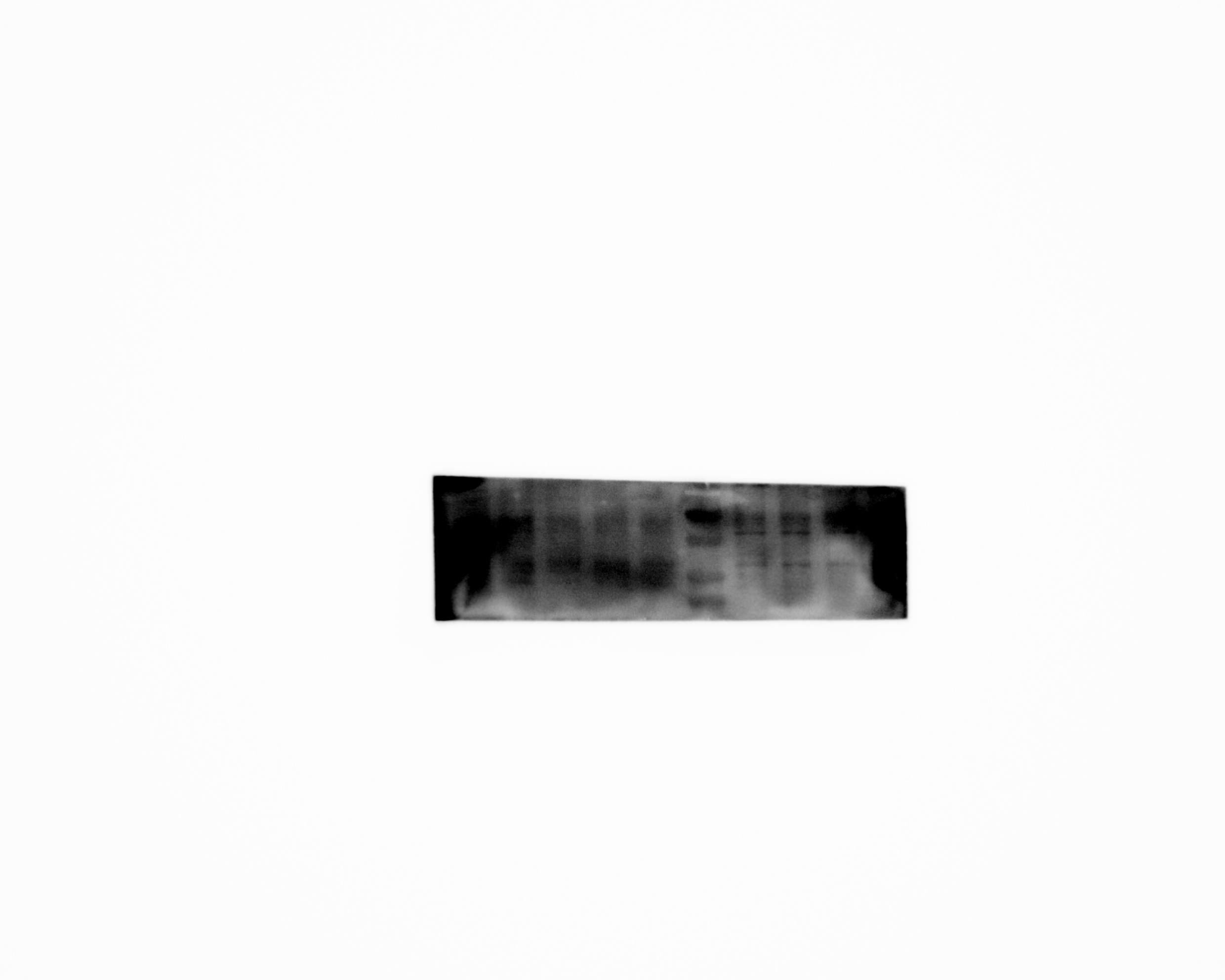

Supplement: Supplementary file 2 — Supplementary materials (Western blot images) [file 41419_2024_7287_MOESM2_ESM.zip › Suppl. materials_WB images/h358 siFTSJ1 actb_2(Chemiluminescence).tif]

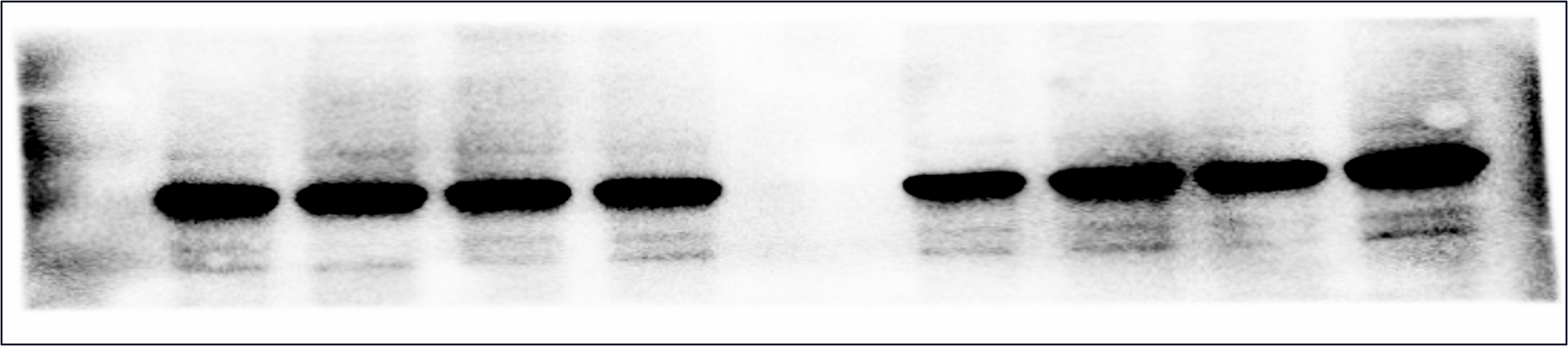

Supplement: Supplementary file 2 — Supplementary materials (Western blot images) [file 41419_2024_7287_MOESM2_ESM.zip › Suppl. materials_WB images/H358 siFTSJ1 PGK1.tif]

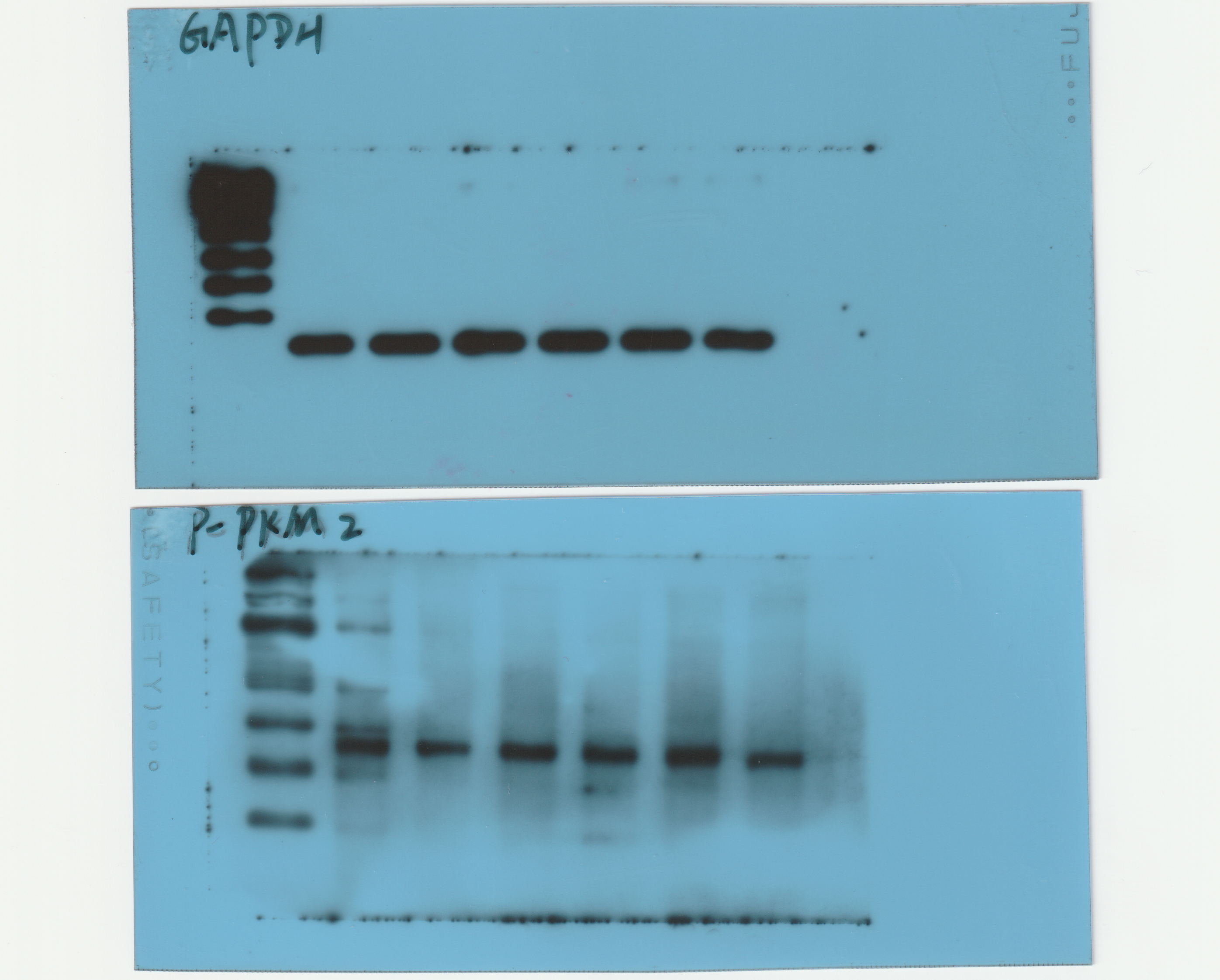

Supplement: Supplementary file 2 — Supplementary materials (Western blot images) [file 41419_2024_7287_MOESM2_ESM.zip › Suppl. materials_WB images/P-PKM2.tif]

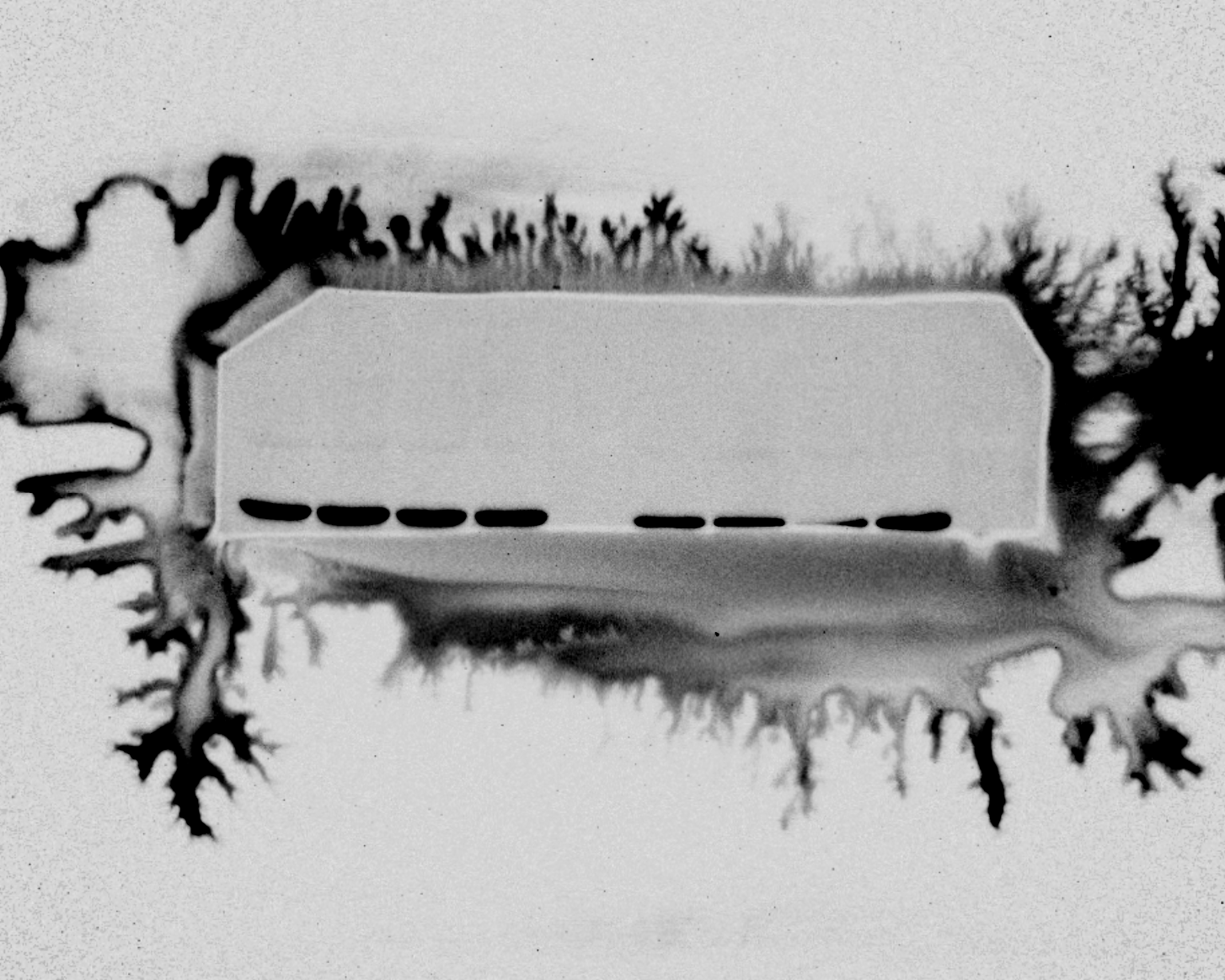

Supplement: Supplementary file 2 — Supplementary materials (Western blot images) [file 41419_2024_7287_MOESM2_ESM.zip › Suppl. materials_WB images/rat ACTB(Chemiluminescence).tif]

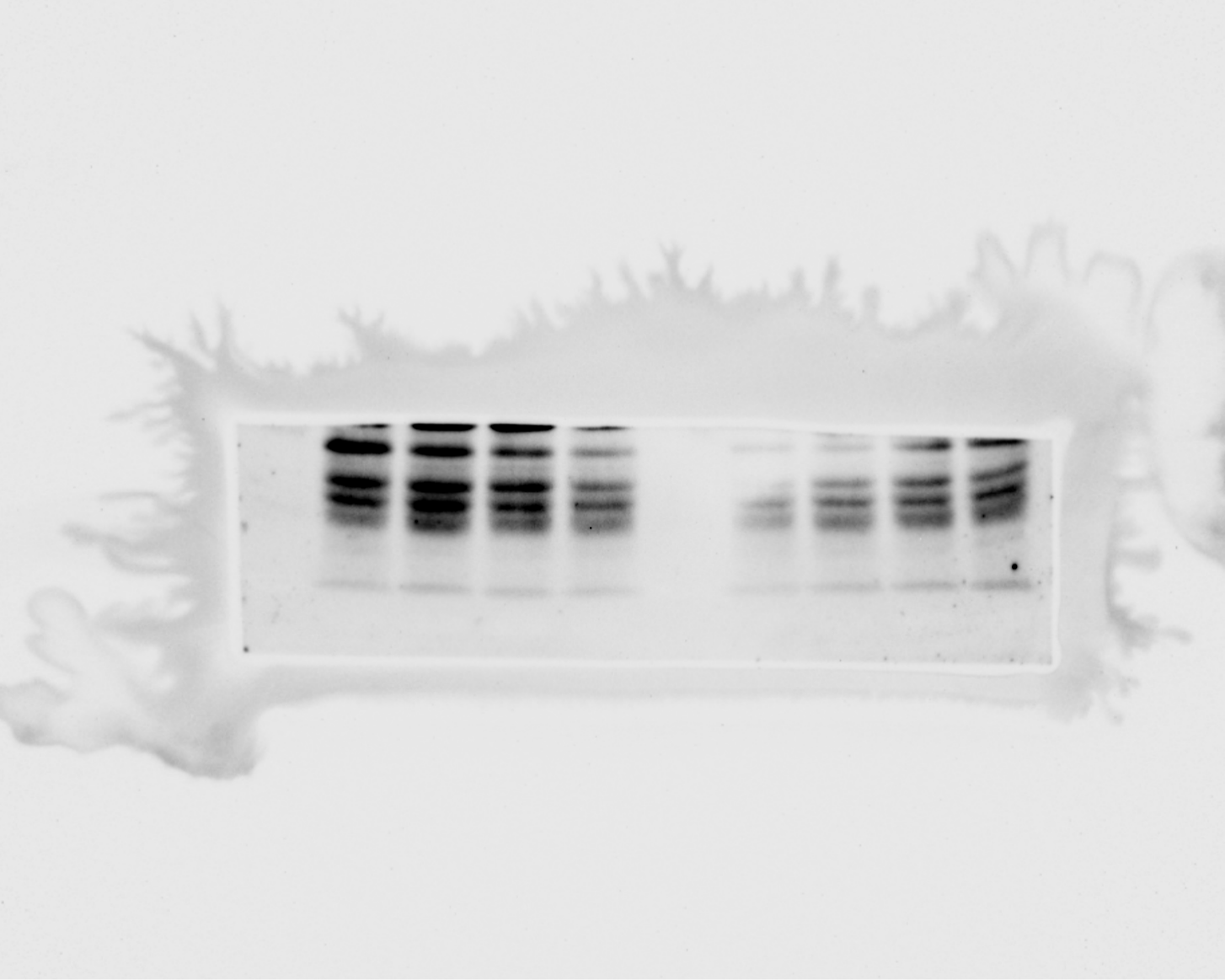

Supplement: Supplementary file 2 — Supplementary materials (Western blot images) [file 41419_2024_7287_MOESM2_ESM.zip › Suppl. materials_WB images/rat ftsj1 (Chemiluminescence).png]
